# Human blood vessel organoids reveal a critical role for CTGF in maintaining microvascular integrity

**DOI:** 10.1038/s41467-023-41326-2

**Published:** 2023-09-09

**Authors:** Sara G. Romeo, Ilaria Secco, Edoardo Schneider, Christina M. Reumiller, Celio X. C. Santos, Anna Zoccarato, Vishal Musale, Aman Pooni, Xiaoke Yin, Konstantinos Theofilatos, Silvia Cellone Trevelin, Lingfang Zeng, Giovanni E. Mann, Varun Pathak, Kevin Harkin, Alan W. Stitt, Reinhold J. Medina, Andriana Margariti, Manuel Mayr, Ajay M. Shah, Mauro Giacca, Anna Zampetaki

**Affiliations:** 1 King’s College London British Heart Foundation Centre, School of Cardiovascular & Metabolic Medicine and Sciences, London, UK; 2 The Wellcome-Wolfson Institute for Experimental Medicine, Queen’s University Belfast, Belfast, UK

**Keywords:** Induced pluripotent stem cells, Extracellular signalling molecules

## Abstract

The microvasculature plays a key role in tissue perfusion and exchange of gases and metabolites. In this study we use human blood vessel organoids (BVOs) as a model of the microvasculature. BVOs fully recapitulate key features of the human microvasculature, including the reliance of mature endothelial cells on glycolytic metabolism, as concluded from metabolic flux assays and mass spectrometry-based metabolomics using stable tracing of ^13^C-glucose. Pharmacological targeting of PFKFB3, an activator of glycolysis, using two chemical inhibitors results in rapid BVO restructuring, vessel regression with reduced pericyte coverage. PFKFB3 mutant BVOs also display similar structural remodelling. Proteomic analysis of the BVO secretome reveal remodelling of the extracellular matrix and differential expression of paracrine mediators such as CTGF. Treatment with recombinant CTGF recovers microvessel structure. In this work we demonstrate that BVOs rapidly undergo restructuring in response to metabolic changes and identify CTGF as a critical paracrine regulator of microvascular integrity.

## Abstract (web summary)

The microvasculature is critical for delivery of oxygen and metabolites throughout tissues. Here they use human blood vessel organoids to show that CTGF is a critical paracrine regulator of microvascular integrity that can restore pericyte coverage and vessel structure.

## Introduction

The microvasculature mediates crucial functions, supplying oxygen and nutrients to tissues, controlling inflammation and exchanging gases and metabolites to and from tissues^1^. Microvessels consisting of endothelial cells (ECs) and pericytes (PCs) form intricate networks in the microvasculature. These two cell types are in direct contact and deposit extracellular matrix (ECM) that forms the basement membrane, with PCs usually fully enclosed in this matrix. Disruption of the EC-PC crosstalk and PC loss from the microvasculature are hallmarks of microangiopathy^2^. Structural remodelling, including changes in vessel density, diameter and length along with alterations of capillary basal membrane thickness, impairs vessel function and can result in tissue hypoxia. In addition, dysregulated PCs display mechanical stiffness and altered contractility that contributes to abnormal EC behaviour, microvascular rarefaction and instability leading to tissue ischaemia^2,3^. Although therapeutic strategies to enhance capillary sprouting and PC lining could stabilise the microvasculature and reduce tissue ischaemia^4,5^, the molecular mechanisms involved in EC and PC dysfunction are not well understood.

Microvascular dysfunction has emerged as a key contributor to cardiovascular diseases. Diabetic microangiopathy is the best characterised microvascular complication of diabetes mellitus^5^, with the duration and severity of hyperglycaemia being important determinants of vascular injury. Recently, the role of functional impairment of the microvasculature has been highlighted as a pathophysiological driver in heart failure^3^. A meta-analysis of seventy-nine studies with a total of ~60,000 individuals concluded that a direct readout of coronary microvascular dysfunction, namely the coronary flow reserve, is strongly associated with increased risk of all-cause mortality and major adverse cardiovascular events across a wide range of pathological processes. Therefore, a diagnostic and prognostic tool of microvascular dysfunction that could facilitate a more in-depth understanding of the disease, could also inform more precise patient selection for personalised therapeutic approaches^6^.

Abnormal angiogenesis in the microvasculature in other organs can also have devastating implications. In the retina, excessive angiogenesis in response to chronic hyperglycaemia and metabolic abnormalities can lead to visual impairment and blindness^7^. Thickening of the basement membrane, disruption of PC- EC interactions and PC apoptosis result in microvascular regression and capillary remodelling with the generation of microaneurysms in the early stages of the disease^7^. In cancer, tumour growth relies on the formation of newly formed vascular networks^8^. These vessels are typically disorganised, tortuous and leaky, lacking normal endothelial and mural cell lining^9^. Initial antiangiogenic therapeutic approaches to target the tumours were unsuccessful as they can induce hypoxia and vessel regression triggering a cascade of events that result in the induction of proangiogenic factors^10^. Treatments aimed to induce tumour vessel normalisation, increase tissue perfusion and improve anti-cancer treatment^11^. Thus, understanding the mechanisms of microvascular homeostasis and identifying the key molecular and cellular regulatory components is important to develop novel therapeutic approaches.

Recently, a human tissue model of the microvasculature has been developed, based on the generation of human blood vessel organoids (BVOs) from induced pluripotent stem (iPS) cells^12^. These self-organising 3D tissue units can be grown in a Petri dish and display the morphological and molecular features of human microvasculature forming capillaries with a lumen, CD31^+^ endothelial lining, platelet-derived growth factor receptor beta (PDGFRβ)^+^ pericyte coverage and the presence of a basement membrane^12^. BVOs derived from human iPS cells are particularly attractive for the development of personalised treatment strategies as they can faithfully recapitulate the human disease features and reflect the genetic heterogeneity of the human population. Thus, iPS-derived BVOs can serve as an experimental system to study the pathogenesis of microvascular dysfunction and as a system for drug and small compound screening^13^.

Metabolism has emerged as a key driver of angiogenesis in parallel to well-established growth factor signalling. In the microvasculature, both ECs and PCs rely heavily on glycolysis for their energy demands^14,15^. Phosphofructokinase-2/ fructose-2,6-bisphosphatase 3 (PFKFB3) is a potent stimulator of glycolysis^16^. In mice, genetic manipulation of PFKFB3 and pharmacological inhibition of PFKFB3 in in vitro experiments resulted in alterations in vascular sprouting and vessel formation^16^, indicating a key role for PFKFB3-driven glycolysis in the angiogenic potential.

Here, we use BVOs to investigate the mechanisms of metabolic rewiring-induced microvascular dysfunction and characterise the response of human microvessels to PFKFB3-driven glycolysis inhibition. Our experiments reveal that CTGF (connective tissue growth factor) is a critical regulator of microvascular integrity. CTGF is a matricellular protein that mediates the interaction between the cell surface and matrix proteoglycans^17^ acting in an autocrine and paracrine manner. Previously, loss of CTGF in mice uncovered an important function of CTGF in the vasculature and vascular defects linked to PC coverage, altered permeability and hypovascularization of the retina^18,19^ while its role in ECM deposition and basal membrane remodelling has linked CTGF to pathological fibrosis^20^ and angiogenesis^21^.

In this work, we demonstrate that supplementation of recombinant CTGF prevents microvascular rarefaction in BVOs following glycolysis inhibition indicating that CTGF could serve as a potential target for therapeutic intervention.

## Results

### Generation of human blood vessel organoids

**Fig. 1:**
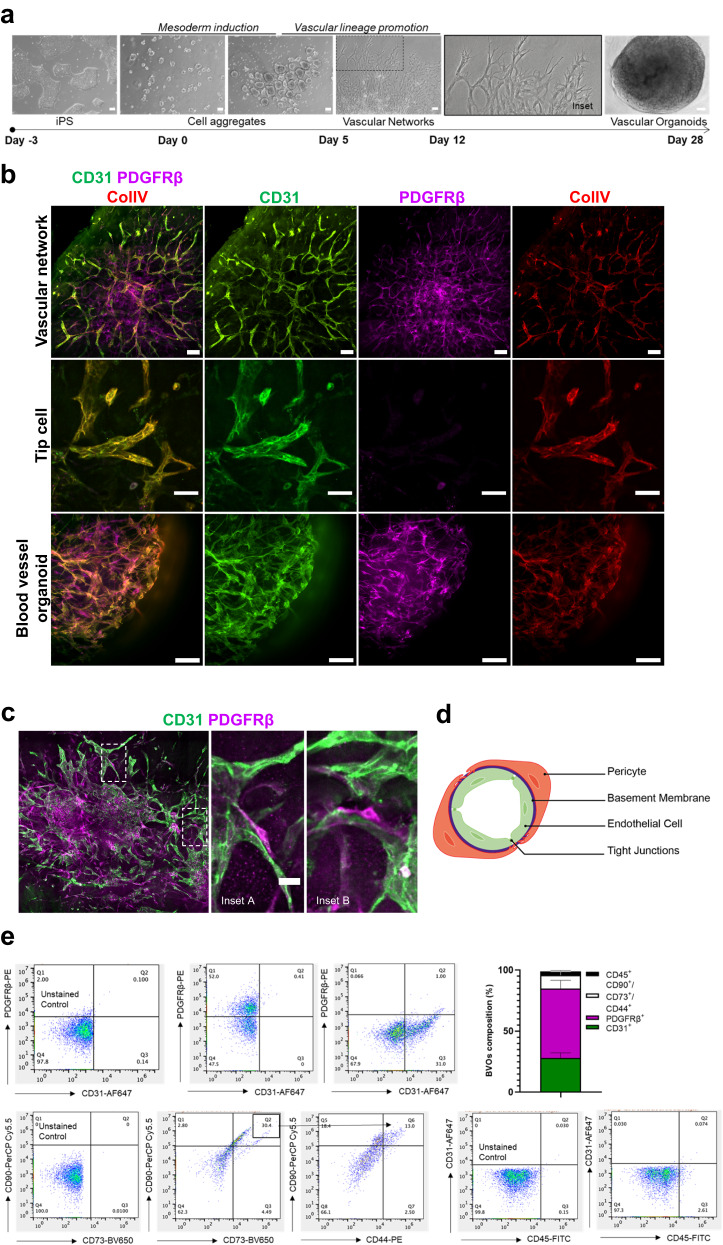
Generation of human blood vessel organoids (BVOs). **a** Bright-field images of human iPS cell differentiation into vascular networks and BVOs. **b** Phenotypic characterisation of vascular networks and BVOs, showing CD31-expressing ECs (green) forming vascular networks covered by PDGFRβ^+^ pericytes (PCs, magenta) and a basement membrane (collagen type IV (Col IV), red). **c** Immunofluorescence confocal imaging of vascular networks showing pericyte coverage (PDGFRβ^+^, magenta) of CD31^+^ ECs (green). **d** Schematic depicting the microvascular structure. **e** FACS was used to determine the different cell populations in BVOs. ECs were defined as CD31^+^, PCs as PDGFRβ^+^, mesenchymal stem-like cells as CD90^+^CD73^+^CD44^+^ and haematopoietic cells as CD45^+^. Cells from a total of 7 BVOs were dissociated and pooled together for this analysis. Data are presented as mean ± SD of *n* = 2 independent experiments. Bar scales 200, 100 and 50 μm. Source data are provided as a Source Data file.

To study the mechanisms of metabolic rewiring-induced microangiopathy, we generated BVOs employing the protocol established by Wimmer et al.^22^. Human iPS cells were used to generate cell aggregates with a diameter ranging from 50 to 150 μm. These cell aggregates subsequently went through mesoderm (days 0–3) and vascular lineage differentiation (days 4–5) and were then embedded in a 3D collagen I-Matrigel matrix in 12-well plates. Vascular networks (VNs) with clearly visible tip cells emerged within 2–3 days. After 5–7 days of sprouting in the 3D substrate, VNs were extracted and transferred into an ultra-low 96-well adhesion plate, where they self-assembled into BVOs (Fig. 1a). BVOs were maintained in suspension culture in a low-attachment 96-well U bottom plate for the duration of the experiment. Immunofluorescence staining was used to characterise the VNs and BVOs and demonstrated the presence of a dense vascular sprouting comprised of CD31^+^ ECs, PDGFRβ^+^ mural cells and a thick deposition of basement membrane as indicated by the detection of Collagen IV (ColIV) in both VNs and whole BVOs (Fig. 1b).

A more detailed analysis of the VN structure revealed PDGFRβ^+^ mural cells tightly associated with ECs, with single PDGFRβ^+^ cells elongated along ECs and embracing the vessel wrapping several ECs incompletely (Fig. 1c) suggesting that these cells are PCs (Fig. 1d). Flow cytometry analysis of digests of BVOs further confirmed the presence of CD31^+^ ECs, mural cells as PDGFRβ^+^ cells, mesenchymal stem-like cells as CD90^+^CD73^+^CD44^+^ cells and haematopoietic cells as CD45^+^ (Fig. 1e and Supplementary Fig. 8). These characteristics indicate that iPS-derived BVOs recapitulate key aspects of the human microvasculature and can be used as a model to study the mechanisms of microangiopathy.

### IPS-derived ECs are highly glycolytic

**Fig. 2:**
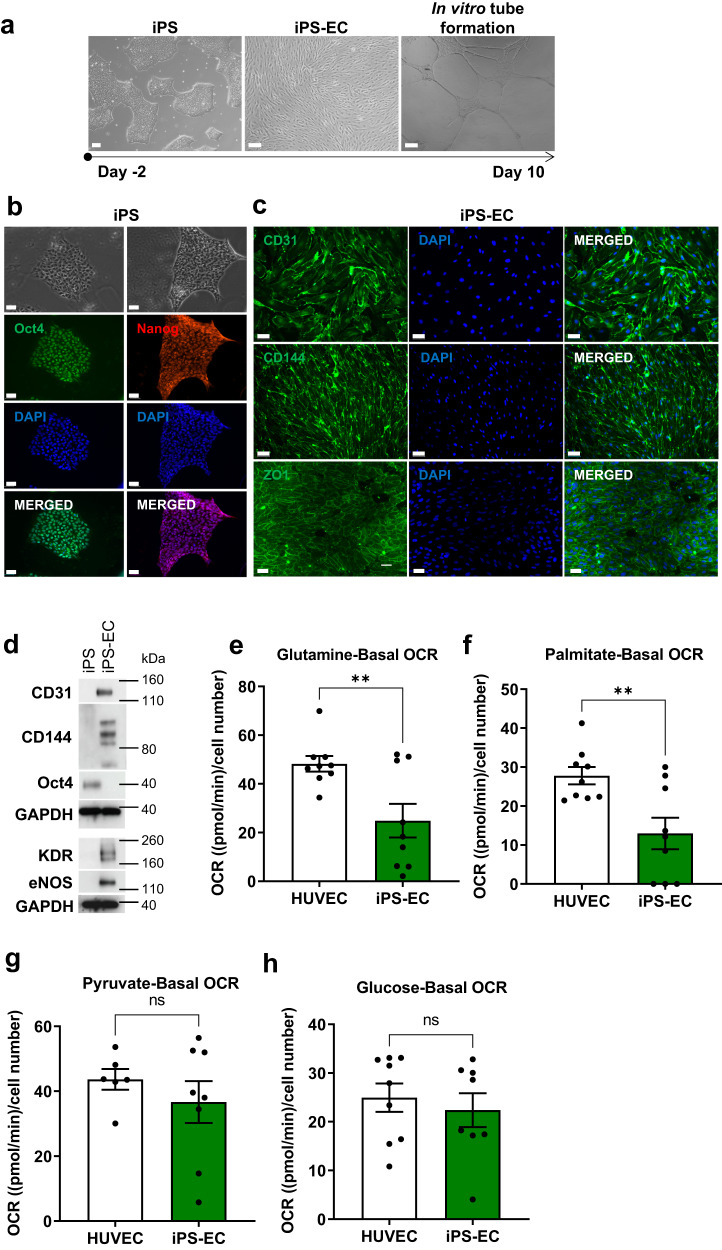
Human iPS cell differentiation toward ECs. **a** Typical morphology of a human iPS colony, iPS derived ECs (iPS-ECs), and iPS-EC in vitro tube formation indicating their angiogenic potential are shown by bright field microscopy. Representative images of five independent experiments. **b** Immunofluorescence confocal image demonstrating the expression of pluripotency markers in human iPS cells and (**c**) EC-specific markers CD31, CD144 and ZO1 in iPS-ECs. Representative images of five independent experiments. **d** The expression of EC markers was confirmed by Western blot. *N* = 2 independent experiments were performed. Quantification of Basal Oxygen Consumption Rate (OCR) measured in iPS-ECs and HUVEC provided with either (**e**) glutamine, (**f**) palmitate, (**g**) pyruvate, or (**h**) glucose in the assay media. Three independent lines were assessed in *n* = 3 wells per assay per line. Values are presented as mean ± SEM; P values were calculated using a two-tailed Student’s *t*-test. (**e**: **p = 0.0072; **f**: **p = 0.0054). ns not significant. Source data are provided as a Source Data file.

Vessel sprouting and angiogenesis are directly linked to the metabolic profile of ECs^15^. Mature ECs rely on glycolysis rather than oxidative phosphorylation for energy production^16^. Aiming to determine whether ECs differentiated from iPS cells have a similar functional and metabolic profile as mature ECs, we generated iPS-derived ECs using a stepwise differentiation protocol to obtain mesodermal progenitors and subsequently vascular lineage cells^23^. CD144^+^ cells from this population were isolated using MACS and their angiogenic potential was tested in a tube formation assay (Fig. 2a). Immunofluorescence staining confirmed successful differentiation, detecting pluripotency markers in iPS cells (Fig. 2b) and the EC markers CD31, CD144 and tight junction protein 1 (ZO1) in the iPS-EC cells (Fig. 2c). These results were also confirmed by western blot analysis (Fig. 2d).

Endothelial glycolytic metabolism has emerged as a major determinant of the angiogenic process^15^. Hence, we evaluated the metabolic profile of iPS-ECs. To determine potential differences in substrate oxidation between iPS-ECs and adult ECs (HUVECs), we determined the oxygen consumption rate (OCR) on a Seahorse instrument^24^. While similar levels of pyruvate and glucose mitochondrial oxidation were detected, a lower preference for glutamine and palmitate was observed in iPS-ECs (Fig. 2e–h and Supplementary Fig. 1a–d).

**Fig. 3:**
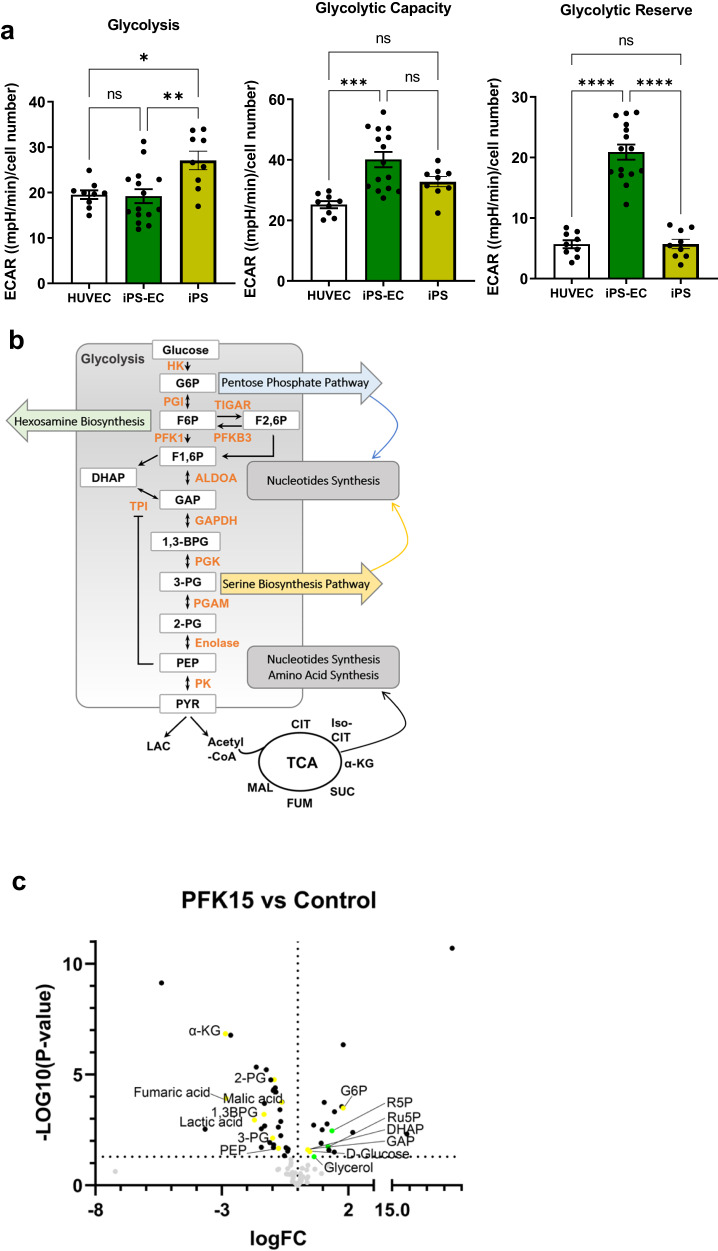
Comparison of glycolytic rates in HUVEC, iPS-ECs and iPS. **a** Glycolysis, glycolytic capacity and glycolytic reserve were measured in HUVEC, iPS-ECs and iPS by performing glycolysis stress tests on a Seahorse XF^e^24. Three independent lines were used for each cell type and *n* = 3 wells for HUVEC and iPS-ECs and *n* = 5 wells for iPS were assessed per line. ECAR (Extracellular Acidification Rate). Data are shown as mean ± SEM using one-way ANOVA followed by Tukey’s multiple comparisons tests. (Glycolysis: *p = 0.0139; **p = 0.0040; Glycolytic Capacity: ***p = 0.0001; Glycolytic Reserve: HUVEC vs. iPS-EC ****p = 0.000000000168; iPS-EC vs. iPS ****p = 0.000000000178). ns not significant. **b** Schematic representation of glycolysis with highlighted metabolic pathways that contribute to biomass production. **c** Volcano plot depicting differences in the metabolite abundance in iPS-ECs following PFK15 treatment for 7 h. Metabolites in glycolysis and the TCA cycle are depicted in yellow, metabolites in the PPP are shown in green. *N* = 3 independent experiments. Statistical comparisons were conducted using the Ebayes method of the limma package. Nominal *p*-values are presented in volcano plot while corrected for multiple testing *p*-values with the Benjamini–Hochberg method are provided in Supplementary Data 1. HK hexokinase, G6P glucose-6-phosphate, PGI phosphoglucose isomerase, F6P fructose-6-phosphate, TIGAR TP53-induced glycolysis and apoptosis regulator, PFKFB3 6-phosphofructo-2-kinase/Fructose-2,6-biphosphatase 3, F-2,6-BP fructose-2,6-bisphosphate, PFK1 phosphofructokinase-1, F1,6 BP fructose-1,6-bisphosphate, ALDOA aldolase A, GAP, G3P glyceraldehyde 3-phosphate, GAPDH glyceraldehyde-3-phosphate dehydrogenase, DHAP dihydroxyacetone phosphate, LAC lactic acid, TPI triosephosphate isomerase, 1,3-BPG 1,3-bisphosphoglycerate, PGK phosphoglycerate kinase, 3-PG 3-phosphoglycerate, PGAM phosphoglycerate mutase, 2-PG 2-phosphoglycerate, PEP phosphoenolpyruvate, PK pyruvate kinase, HBP hexosamine biosynthetic pathway, PPP pentose phosphate pathway, R5P ribose 5-phosphate, Ru5P ribulose 5-phosphate, S7P sedoheptulose 7-phosphate, UDP-GlcNAc uridine diphosphate *N*-acetylglucosamine, P-Serine phosphoserine, α -KG α-Ketoglutaric acid, 2-HG 2-Hydroxyglutarate. Source data are provided as a Source Data file.

To compare the reliance of iPS-ECs and HUVECs on glycolysis for energy production, we quantified the extracellular acidification rate (ECAR) on a Seahorse flux analyzer, as an indication of lactate production. IPS-ECs demonstrated glycolysis, similar to HUVECs and an even higher glycolytic capacity and reserve (Fig. 3a). Interestingly, iPS cells displayed the highest levels of glycolysis compared to the two EC types. Targeted stable isotope tracing of U-^13^C_6_ glucose in HUVECs and iPS-ECs showed similar ^13^C label incorporation levels into glycolytic intermediates (Supplementary Fig. 1e), thereby confirming the observation of comparable glycolysis rates in these two distinct cell types. These data demonstrate that iPS cells differentiated into ECs display key morphological and functional properties of mature ECs.

A key regulator of glycolysis in ECs is PFKFB3, a phosphofructokinase-2/fructose-2,6-bisphosphatase enzyme that synthesises fructose-2,6-bisphosphate (F2,6P2), an allosteric activator of PFK-1, the most potent stimulator of glycolysis (Fig. 3b)^16^. Genetic targeting of PFKFB3 in mice and pharmacological inhibition in in vitro experiments results in alterations in vascular sprouting and vessel formation^16^, indicating a key role for PFKFB3-driven glycolysis in the angiogenic process.

In human iPS-derived ECs, we found that PFK15^25^, a chemical inhibitor of PFKFB3 does not affect cell survival of iPS-ECs up to a concentration of 5 μM (Supplementary Fig. 2). Treatment of iPS-ECs with PFK15 (2.5 μM) had a profound effect on the metabolic profile of the cells, with a significant decrease in metabolites related to glycolysis (2-PG, 3-PG, GAP, Lactate) and the tricarboxylic acid (TCA) cycle (α-KG, Malic acid, Fumaric acid) whereas there was a significant increase in metabolites related to the pentose phosphate pathway and nucleotide synthesis (R5P, Ru-5-P; Fig. 3c and Supplementary Data file 1).

### Inhibition of glycolysis and BVOs cellular composition

Aiming to go beyond the response of a single cell type and delineate the impact of short-term PFKFB3 inhibition in the human microvascular tissue, we employed BVOs for further studies. BVOs represent a human model of self-assembled 3D microvascular tissue that can offer unique insights into the mechanisms of tissue remodelling. It can overcome interspecies variations and is more physiologically relevant to co-culture systems. Additionally, BVOs can be used to determine cell-to-cell and cell-to-ECM interactions, hence providing a better model of the human microvasculature^13,26^.

After a 24 h PFK15 treatment, no modification in BVO diameter or organoid disintegration was observed (Supplementary Fig. 3a, b). This is important as changes in organoids size have been previously linked to their function^27^. Although no significant difference in cell numbers in the treated and untreated organoids was detected, inhibition of glycolysis led to a significant decrease in the number of proliferating cells accompanied by an increase in apoptosis (Supplementary Fig. 3c–e). These findings indicate that inhibiting PFKFB3-driven glycolysis in BVOs leads to alteration in the cellular state, but not to organoid disintegration.

### Inhibition of glycolysis triggers restructuring of BVOs

**Fig. 4:**
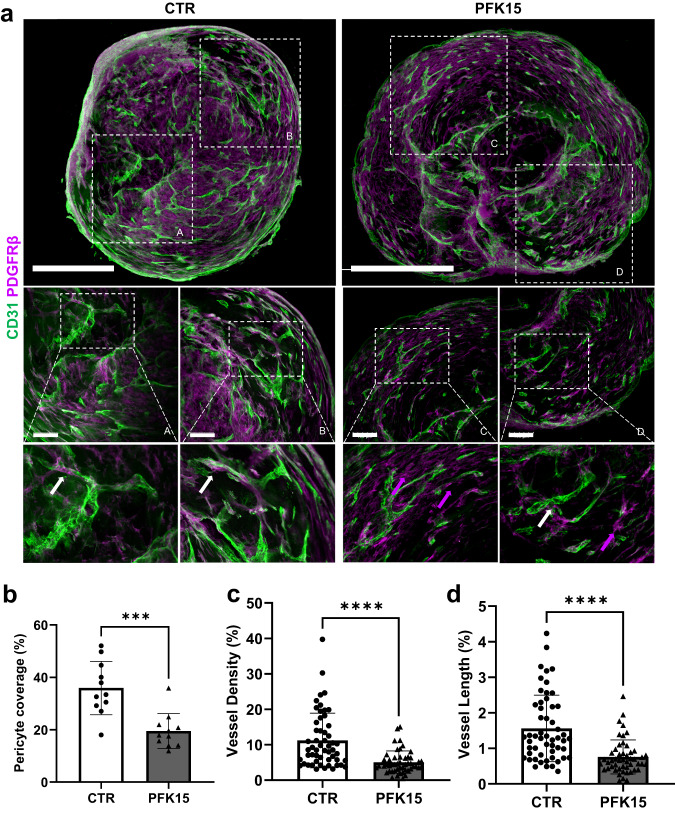
The effect of PFK15 on BVO structure. **a** Immunofluorescence confocal imaging of BVO sections showing pericyte coverage (PDGFRβ, magenta) of CD31^+^ ECs (green). White arrows indicate pericytes attached to microvessels, magenta arrows indicate extravascular mural cells. **b** Percentage of pericyte coverage *n* = 6 BVOs per group, from three separate preparations. One-two sections per BVO were assessed. **c** Quantification of vessel density, (**d**) length in *n* = 6 BVOs per group, from three separate preparations in four different areas per x10 images. One-two sections per BVO were assessed. Values are presented as mean ± SD; *P*-values were calculated using a two-tailed Student’s *t*-test. (**b**: ***p = 0.0002; **c**: ****p = 0.000000894; **d**: ****p = 0.000000394). Bar scales 500 and 50 μm. Source data are provided as a Source Data file.

BVOs display striking similarity with the human microvasculature, consisting of a dense network of capillaries with CD31^+^ endothelial microvessels with PDGFRβ^+^, chondroitin sulfate proteoglycan 4 (NG2)^+^ mural cells and pericyte coverage of microvessels (Fig. 4a, Supplementary Fig. 4a and supplementary movies 1 and 2). Interestingly, even a short 24 h inhibition of PFKFB3-driven glycolysis led to structural modification of BVOs and resulted in reduced pericyte coverage by >50% (Fig. 4a, b). In PFK15-treated BVOs, pericyte dropout was observed with a significant portion of PDGFRβ^+^ cells becoming extravascular and not attached to CD31^+^ ECs. To determine the presence of fibroblasts in BVOs, PDGFRα expression was used as an independent marker. Very few PDGFRα positive cells that did not change upon PFK15 treatment were detected (Supplementary Fig. 4b). Additionally, quantification of microvessels revealed that in PFK15-treated BVOs, both vessel density and vessel length (Fig. 4c, d) decreased significantly.

**Fig. 5:**
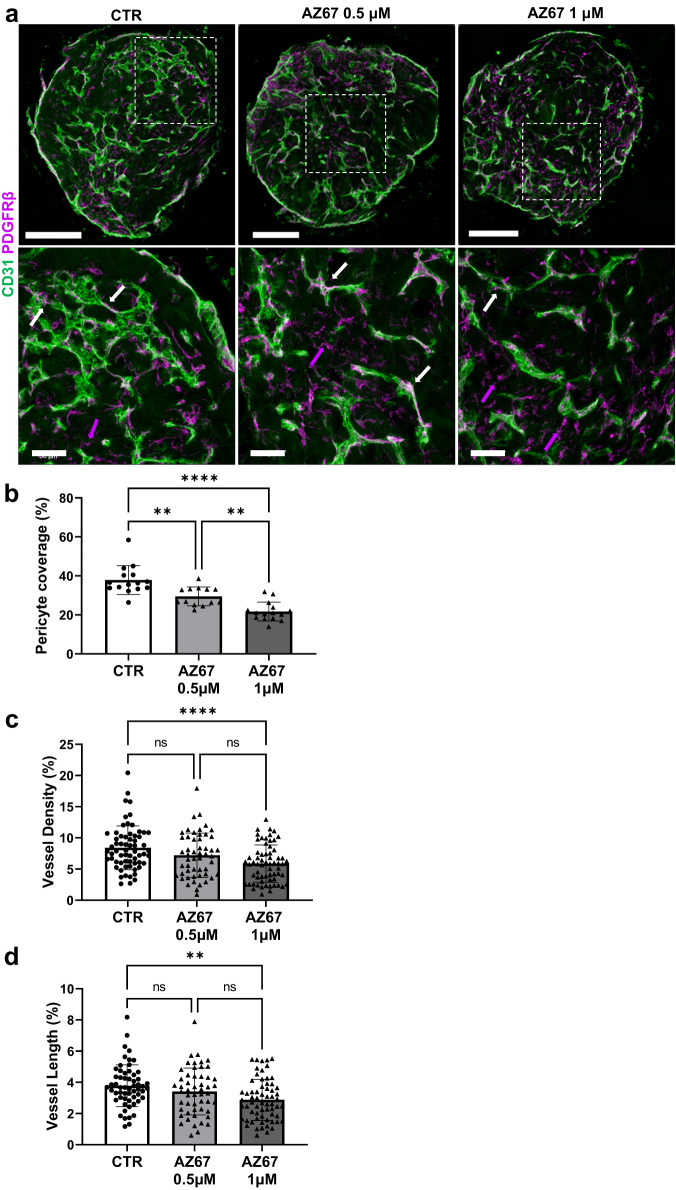
The effect of AZ67 on BVO structure. **a** Immunofluorescence confocal imaging of BVO sections showing pericyte coverage (PDGFRβ, magenta) of CD31^+^ ECs (green). **b** Percentage of pericyte coverage *n* = 7 BVOs per group, from three separate preparations. White arrows indicate pericytes attached to microvessels, magenta arrows indicate extravascular mural cells. One-two sections per BVO were assessed. **c** Quantification of vessel density, **d** length in *n* = 7 BVOs per group, from 3 separate preparations in 4 different areas per x10 images. One-two sections per BVO were assessed. Values are presented as mean ± SD; *P* values were calculated using a one-way ANOVA followed by Tukey’s multiple comparisons tests. (**b**: CTR vs. AZ67 0.5 μM **p = 0.0015; CTR vs. AZ67 1 μM ****p = 0.000000010; AZ67 0.5 μM vs. AZ67 1 μM **p = 0.0034; **c**: **** *p* = 0.000096; **d**: **p = 0.0012). ns not significant. Bar scales 200 and 50 μm. Source data are provided as a Source Data file.

These findings were also confirmed using compound AZ67, a new generation, potent and selective PFKFB3 inhibitor^28^. Similar to PFK15 treatment, AZ67 decreased pericyte coverage and increased extravascular PDGFRβ^+^ mural cells (Fig. 5a, b). BVOs treated with AZ67 displayed structural remodelling, with reduced vessel density and vessel length (Fig. 5c, d). These findings demonstrate that, in response to alteration in glycolysis, BVOs undergo rapid structural remodelling and microvessel regression.

Hypoxia is a common feature in many pathological conditions, including cardiovascular and retinal disease and cancer^29,30^. To determine whether exposing BVOs to hypoxia can trigger structural remodelling, we incubated BVOs in 1% O_2_ for 24 h. Similar to the chemical inhibitors of PFKFB3, a drop in pericyte coverage accompanied by a decrease in vessel density and length was observed in hypoxic BVOs, compared to normoxic controls (Supplementary Fig. 5).

### Genetic deletion of *PFKFB3* triggers remodelling of BVOs

To determine the effect of genetic targeting of *PFKFB3* in the human microvasculature, we took advantage of the CRISPR-Cas9 genome editing method to generate *PFKFB3* knockout iPS clones (Supplementary Fig. 6).

**Fig. 6:**
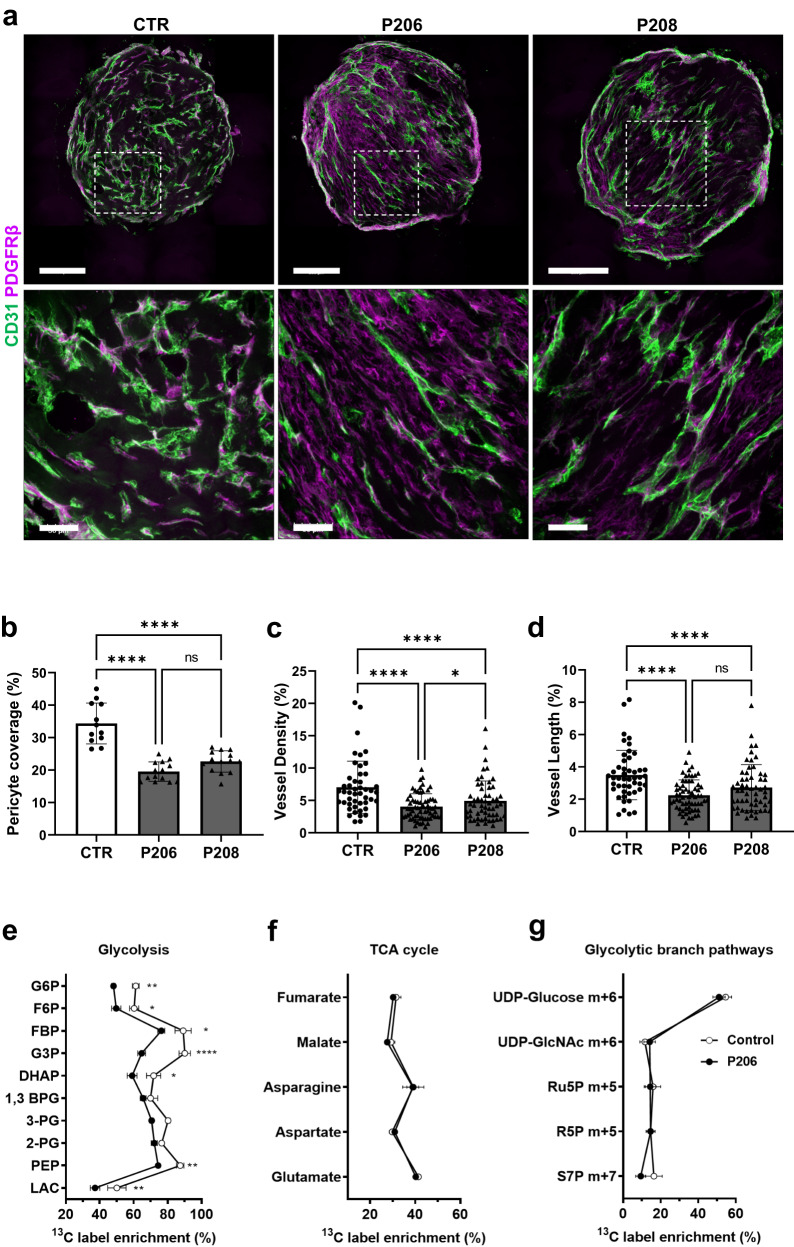
The effect of *PFKFB3* knockout on BVO structure. **a** CRISPR-Cas9 genome editing was used to generate *PFKFB3* knockout iPS cells. Immunofluorescence confocal imaging of unedited control (CTR) and *PFKFB3* knockout (P206, P208) BVO sections showing pericyte coverage (PDGFRβ, magenta) of CD31^+^ ECs (green). **b** Percentage of pericyte coverage CTR *n* = 6 BVOs, P206 *n* = 7 BVOs, P208 *n* = 7 BVOs, from three separate preparations. One-two sections per BVO were assessed. **c** Quantification of vessel density and (**d**) length in CTR *n* = 6 BVOs, P206 *n* = 7 BVOs, P208 *n* = 7 BVOs, from three separate preparations in four different areas per x10 images. One-two sections per BVO were assessed. Values are presented as mean ± SD using two-way ANOVA followed by Tukey’s multiple comparisons tests. (**b**: CTR vs. P206 ****p = 0.000000000522; CTR vs. P208 ****p = 0.000000128; **c**: *p = 0.0374; CTR vs P206 ****p = 0.000027; CTR vs P208 ****p = 0.000000022; **d**: CTR vs P206 ****p = 0.000088; CTR vs P208 ****p = 0.000005). ns not significant. Bar scales 200 and 50 μm. Effect of PFKFB3 knockout on ^13^C label incorporation into metabolites related to (**e**) glycolysis, (**f**) the TCA cycle and (**g**) glycolytic branch pathways after 3 h of incubation with ^13^C_6_-glucose. Data represents mean ± SEM, *n* = 4 independent experiments, statistical significance was assessed by a two-way ANOVA with Holm-Sidak post-hoc test. (**e**: LAC **p = 0.00618; PEP **p = 0.00531; DHAP *p = 0.02990; G3P ****p = 0.000000798; FBP *p = 0.02749; F6P *p = 0.04479; G6P*p = 0.00473). G6P glucose 6-phosphate, F6P fructose 6-phosphate, FBP fructose 1,6-bisphosphate, G3P glyceraldehyde 3-phosphate, DHAP dihydroxyacetone phosphate, 1,3-BPG 1,3-bisphosphoglycerate, 2-PG 2-phosphoglycerate, PEP phosphoenolpyruvate, LAC lactate, UDP uridine diphosphate, GlcNAc *N*-acetylglucosamine, Ru5P ribulose 5-phosphate, R5P ribose 5-phosphate, S7P sedoheptulose-7-phosphate, m + x mass isotopologues x. Source data are provided as a Source Data file.

Two separate isogenic iPS mutants (labelled as P206 and P208) and their parental iPS line were then used to generate BVOs. Genetic targeting of glycolysis led to structural remodelling, with mutant BVOs displaying a diminished vascular network in terms of vessel density and length. This was accompanied by low pericyte coverage (Fig. 6a–d). Metabolic flux analysis of ^13^C-glucose in BVOs indicated a profound change in glucose utilisation upon *PFKFB3* knockout (Fig. 6e–g). There was a significant reduction in ^13^C label incorporation from glucose into glycolytic intermediates up- and downstream of PFKFB3 (Fig. 6e) in *PFKFB3* knockout BVOs. Interestingly, the disruption in glycolysis did not affect the glucose contribution to the TCA cycle (Fig. 6f) and glycolytic branch pathways (Fig. 6g).

### BVO secretome analysis reveals a complex ECM deposition

Maintaining microvascular integrity requires tight regulation of the EC-PC interactions. In addition to direct contact^14^, this crosstalk also relies on the secretion of paracrine regulators^31^. BVOs are self-assembled microvascular tissue units that represent an excellent human model to study EC: PC interactions as BVOs are devoid of the major limitations of in vitro 2D cell culture and co-culture systems^26^. Therefore, to identify critical paracrine mediators we performed a proteomic analysis of the secretome of BVOs at baseline and following PFK15 treatment. Only proteins reported at the MatrisomeDB plus some additional secreted proteins from our in-house generated database were considered. Using the signalP tool, 87.25% of these proteins are found to be classified as secreted based on the presence of a signal peptide (Supplementary Data Files 2–4).

**Fig. 7:**
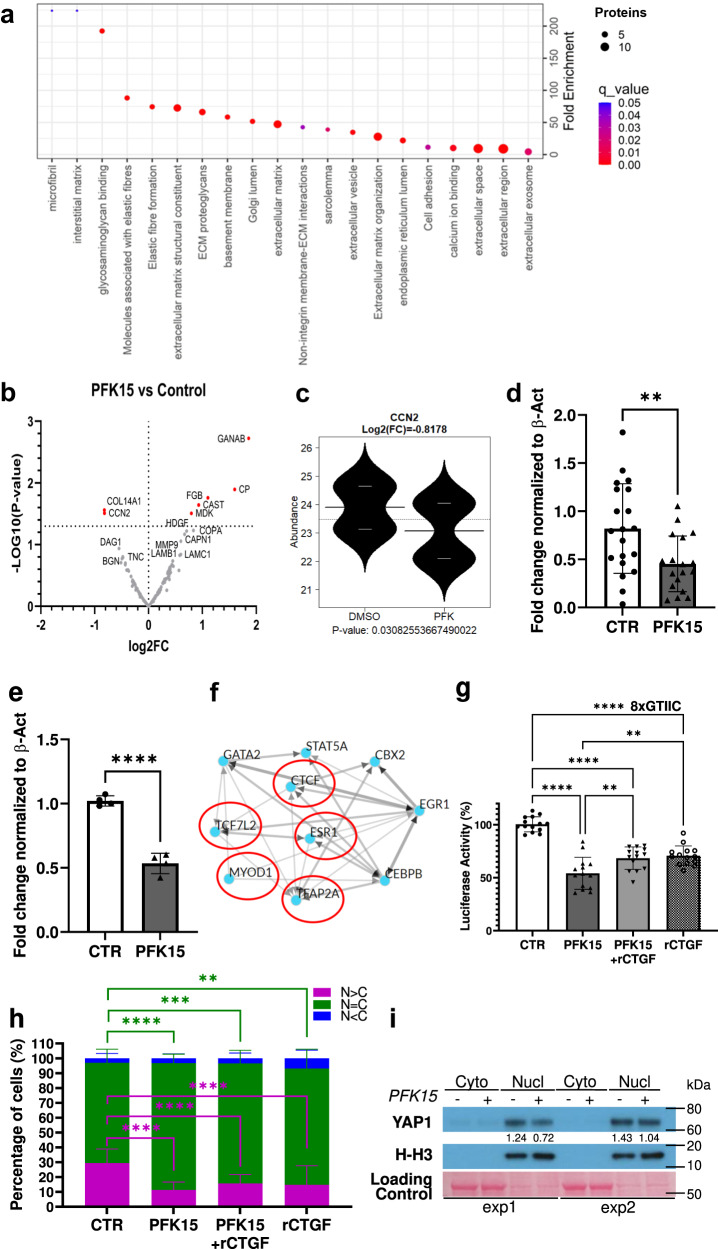
Proteomic analysis of the BVO secretome. **a** Pathway enrichment and (**b**) volcano plot of proteins detected in the secretome of BVOs treated with DMSO (CTR) or PFK15 (2.5 µM) for 24 h. *n* = 5 BVOs per pooled sample, from 2 separate preparations. *P*-values < 0.05 shown in red. Volcano plot was generated using GraphPad Prism 9 software and statistical comparisons were conducted using the Ebayes method of the limma package. Nominal *p*-values are presented in volcano plot while corrected for multiple testing *p*-values with the Benjamini-Hochberg method are provided in Supplementary Data 2. **c** CTGF expression in the BVOs secretome, *n* = 5 BVOs per pooled sample, from 2 separate preparations. Statistical comparison was conducted using the Ebayes method of the limma package. Nominal p-value is displayed in beanplot while corrected for multiple testing *p*-value with the Benjamini-Hochberg method is provided in Supplementary Data 2. **d** QPCR quantification of *CTGF* expression in BVOs (*n* = 20 per group from three separate preparations) and in iPS-ECs (**e**) (*n* = 4 independent preparations). Data are shown as mean ± SD; *p* values were calculated using a two-tailed Student’s *t*-test (BVOs: **p = 0.0068, iPS-ECs: ****p = 0.000034). β-actin was used as a normalisation control. **f** Transcription factor enrichment analysis for differentially expressed proteins using the ChEA3 tool. Red circles indicate putative binding partners of YAP. **g** YAP reporter activity in HEK293T cells cultured as indicated for 24 h. rCTGF: recombinant CTGF. Data from three independent transfections in quadruplicates are shown as mean ± SD using two-way ANOVA followed by Tukey’s multiple comparisons tests. (CTR vs. PFK15 ****p = <0.000000000000001; CTR vs. PFK15 +rCTGF ****p = 0.000000002; CTR vs. rCTGF ****p = 0.000000013; PFK15 vs. PFK15+rCTGF **p = 0.0057; PFK15 vs. rCTGF **p = 0.0010). YAP subcellular localisation in iPS-EC after 3 h treatment with PFK15 as detected using (**h**) immunofluorescence confocal microscopy. N nucleus, C cytosol. *n* = 400 cells per group. Data are shown as mean ± SD using two-way ANOVA followed by Tukey’s multiple comparisons tests. (N > C: CTR vs. PFK15 ****p = 0.000000138; CTR vs. PFK15 +rCTGF ****p = 0.000064; CTR vs. rCTGF ****p = 0.000020; *N* = C: **p = 0.0030; ***p = 0.0001; ****p = 0.000000216) and (**i**) western blot analysis. Nucl nucleus, Cyto cytosol. Histone 3 expression (H-H3) was a normalisation control for the nuclear fraction. Loading control: Ponceau staining. Two independent experiments were performed. Source data are provided as a Source Data file.

A total of 149 proteins were identified in the conditioned media of BVOs with the top gene ontology pathway terms associated with ECM deposition (Fig. 7a and Supplementary Data 2). A rich ECM comprising 58 structural and matricellular proteins and a network of 32 proteases, protease inhibitors and ECM degrading enzymes were detected. This indicated the presence of an intricate dynamic basement membrane that can adapt and respond to stimulation (Fig. 7b).

The detected upregulation of the matrix-degrading enzyme matrix- metalloproteinase 9 (MMP9) in PFK15-treated BVOs (Supplementary Fig. 7a) was particularly interesting. Quantitative PCR analysis in BVOs revealed a sharp increase of MMP9 mRNA following PFK15 treatment (Supplementary Fig. 7b) and zymography confirmed enhanced enzymatic activity of gelatinases in the PFK15-treated BVO conditioned media (Supplementary Fig. 7c). In line with the reported inverse correlation between MMP9 and ColIV^32^, in PFK15-treated BVOs the increased MMP9 activity coincided with reduced ColIV in the basement membrane (Supplementary Fig. 7d, e). These findings are consistent with the presence of a highly complex basement membrane in BVOs, with selected ECM structural and matricellular proteins being deposited and degraded in response to metabolic rewiring.

### PFKFB3-driven glycolysis inhibition and CTGF expression

Our proteomic analysis revealed that the cellular communication network factor 2 (CCN2, also known as CTGF), and a potent mitogen secreted by ECs^21^, were among the most downregulated proteins in the secretome of PKF15 treated BVOs (Fig. 7b, c). In line with the proteomic data, reduced *CTGF* mRNA was detected by qPCR in PFK15-treated BVOs (Fig. 7d) and iPS-ECs (Fig. 7e).

CTGF expression is often used as a read-out of YAP (yes-associated protein 1) activity^33^. YAP and TAZ (transcriptional co-activator with PDZ-binding motif) are two transcriptional coactivators that are retained in the cytosol when rendered inactive by phosphorylation while, upon activation, translocate to the nucleus. Here, they bind the TEA domain DNA-binding transcription factors and initiate a cascade of events to regulate angiogenesis by controlling the EC proliferation, junction assembly and migration^34,35^. YAP has emerged as an important mechano-transduction sensor that may also be sensitive to metabolic changes in the cells^36,37^. In our proteomic dataset, transcription factor enrichment analysis revealed a regulatory network consisting of 10 transcription factors that control differentially expressed genes (Fig. 7f). Among these genes, five transcription factors are potential binding partners of YAP^38^.

Consistent with an involvement of YAP in this process, YAP reporter assays indicated a decrease in YAP activity following PFK15 treatment (Fig. 7g). Recombinant CTGF supplementation could partially restore YAP activity in PFK15-treated cells (Fig. 7g). Immunofluorescence experiments revealed that PFK15 treatment of iPS-ECs lowered the levels of YAP in the nuclear fraction, which was evident after 3 h of treatment (Fig. 7h). This finding that was also confirmed by western blot analysis (Fig. 7i). These data highlight the presence of a CTGF-YAP feedforward loop in maintaining vascular integrity.

### CTGF can recover tight junction morphology

**Fig. 8:**
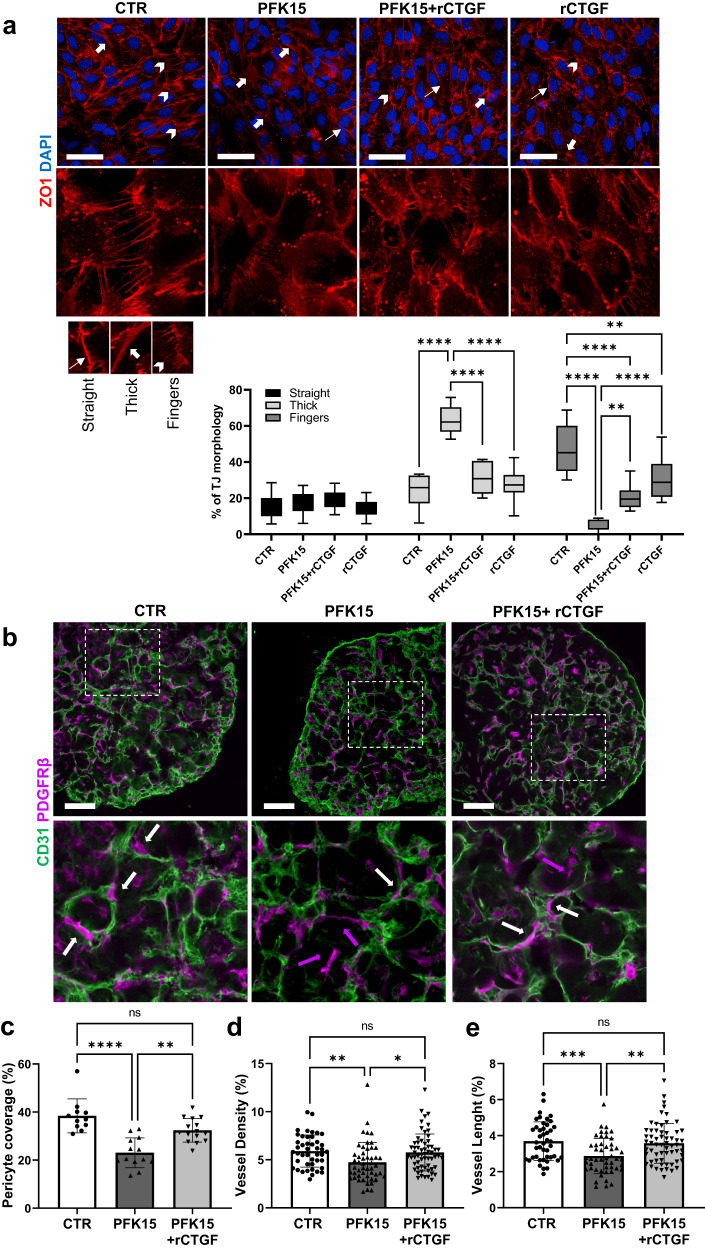
CTGF can restore vascular integrity. **a** Phenotypic characterisation of tight junction morphology in iPS-ECs treated as indicated for 3 h using immunofluorescence confocal imaging. Lower panel, quantification of straight, thick and fingers junctions. *n* = 250 cells per group. Median values are shown in each boxplot. All boxes include the median line, and the box denotes the interquartile range (IQR). Whiskers denote the rest of the data distribution and spread from minimum to maximum. All p values were calculated using a using a two-way ANOVA followed by Tukey’s multiple comparisons tests. (Thick: CTR vs. PFK15 ****p < 0.000000000000001; PFK15 vs. PFK15+rCTGF ****p = 0.000000016; PFK15 vs. rCTGF ****p = 0.000000000017 Fingers: CTR vs. PFK15 ****p < 0.000000000000001; CTR vs. PFK15+rCTGF ****p = 0.000002; CTR vs. rCTGF **p = 0.0035; PFK15 vs. PFK15+rCTGF **p = 0.0043; PFK15 vs. rCTGF ****p = 0.000000781). **b** Immunofluorescence confocal imaging showing CD31^+^ ECs (green) in vascular networks covered by pericytes (PDGFRβ^+^, magenta) in sections from BVOs treated with DMSO (CTR) or PFK15 (2.5 µM) and PFK15 (2.5 µM) + rCTGF(50 ng/ml) for 24 h. White arrows indicate pericytes attached to microvessels, magenta arrows indicate extravascular mural cells. **c** Pericyte coverage in *n* = 6 BVOs per group from three separate preparations. One-two sections per BVO were assessed. **d** Quantification of vessel density and (**e**) length in *n* = 6 BVOs per group from three separate preparations and 4 different areas per 10x images have been used. One-two sections per BVO were assessed. Data are shown as mean ± SD using two-way ANOVA followed by Tukey’s multiple comparisons tests. (**c**: ****p = 0.000061; **p = 0.0058; **d**: *p = 0.0150 **p = 0.0097; **e**: **p = 0.0019 ***p = 0.0008). ns not significant. Bar scales 100 μm. Source data are provided as a Source Data file.

The formation of adhesion plaques and gap junctions plays a key role in the crosstalk between ECs and PCs^39^. ZO1 is a structural adaptor protein that coordinates the binding of F-actin and the transmembrane and cytosolic proteins that are required for tight junction function^34,39^. Following the protocol described by Neto et al.^34^ for tight junction quantification, we found that PFK15 treatment in iPS-ECs modified the junctional morphology with an increase in thick junctions, typically linked with increased permeability and a reduction in fingers junctions, which are associated with tip cells and migration^34^ (Fig. 8a). Furthermore, recombinant CTGF abolished the increase in thick junctions in the PFK15-treated iPS-ECs and led to a partial recovery of fingers junctions (Fig. 8a). These findings highlight the impact of PFKFB3 inhibition on YAP and its downstream effector CTGF in BVO structural remodelling and indicate an important role for CTGF in maintaining tight junction morphology.

### CTGF and BVO restructuring following PFK15 treatment

To determine whether the PFK15-induced remodelling in BVOs can be reversed by CTGF, recombinant CTGF was used as a supplement in PFK15-treated BVOs. The self-assembled 3D vascular tissue showed again a well-organised interconnected capillary network consisting of ECs and tightly associated PCs in the control BVOs. As expected, PFK15 treatment diminished the vascular network with reduced vessel density and length accompanied by low pericyte coverage (Fig. 8b–e). Recombinant CTGF could abrogate the PFK15-triggered structural remodelling in BVOs. BVOs treated with PFK15 in the presence of recombinant CTGF displayed a robust and highly significant increase in PC coverage in recombinant CTGF-treated PFK15 BVOs (Fig. 8c). This coincided with vessel density and length similar to control BVOs and higher vessel density and length compared to the PFK15 BVOs (Fig. 8d, e). These findings demonstrate that CTGF plays a key role in PC recruitment to capillaries and the maintenance of microvascular integrity.

## Discussion

Here, we show that human iPS-derived BVOs respond rapidly and undergo structural remodelling after short pharmacological inhibition of PFKFB3-driven glycolysis. Vessel regression, as highlighted by a reduction in vessel density and length as well as by changes in microvasculature proliferation and apoptosis, was readily detectable in BVOs even after a short 24 h drug treatment. Furthermore, proteomic analysis of the BVO secretome revealed paracrine mediators implicated in ECM deposition and remodelling and identified CTGF as a critical regulator of microvascular integrity.

In our experiments, PFKFB3-driven glycolysis has emerged as a key metabolic pathway in a human model of the microvasculature. Previous studies have underlined the highly glycolytic nature of ECs and have highlighted the advantages of using aerobic glycolysis over oxidative metabolism in the EC angiogenic potential^16^.

Here, by employing extracellular flux analysis and stable isotope tracing of U-^13^C_6_ glucose labelling, we demonstrate that iPS-ECs also rely heavily on glycolysis (versus oxidation), providing further support to the notion that human iPS-derived vascular tissue can be used as a model to faithfully recapitulate the properties and responses of the human vasculature. The metabolic rewiring induced by targeting PFKFB3-driven glycolysis in BVOs, triggered a cascade of events that had a profound effect on both the structural components of the microvasculature, namely ECs, PCs and basement membrane- and their interactions.

Similar to the *PFKFB3* deletion in ECs in mice^16^, we observed impaired expansion of vascular plexus in PFK15-treated BVOs. A significant decrease in vessel density and length was noted following glycolysis inhibition in BVOs. In terms of cell localisation, we detected a pronounced PC dropout (~50%) in human PFK15-treated BVOs, contrary to the findings in mice harbouring a deletion of *PFKFB3* in ECs, where no changes were detected^16^. Likewise, results obtained by CRISPR-Cas9 genetic targeting of *PFKFB3*, showed that BVOs lacking *PFKFB3* expression displayed reduced PC coverage. Although the mechanism is currently unclear, disruption of *PFKFB3* in both mural cells and ECs in BVOs and interspecies differences (human versus mouse) could explain the different phenotypes.

The tight interactions between ECs and PCs are critical for the integrity of mature capillary networks and the detachment of PCs from the microvasculature has a destabilising effect on microvessels^40^. Noteworthy, pioneering studies in *PDGFβ*+/- mice demonstrated that a 50% reduction in PC density can have a causal effect in triggering pathological angiogenesis^4^. In PFK15-treated BVOs the 50% decline in PC coverage was accompanied by microvascular regression with reduced vessel density and length. Even though the sequence of these events is not clear, the proteolytic degradation of the basement membrane by MMPs has been implicated in the detachment of PCs^41^. Hence, the increased presence and proteolytic activity of MMP9 in the PFK15 BVOs as confirmed by proteomic analysis and zymography may facilitate the increased release of PCs from the basement membrane, a concept that is also supported by the reduced thickness of ColIV in the PFK15-treated microvessels.

A mechanistic link between ECM and glycolysis has been previously reported^42^. ECM stiffness was shown to play a key role in regulating cellular glucose uptake, glycolysis and glycogen synthesis by affecting Rho/Rock-actin cytoskeleton. Glycolysis responds to alteration of the actomyosin cytoskeleton, thus coupling cell metabolism to the mechanical properties of the ECM. These processes can vary in normal and tumour cells and cytoskeleton resistance to mechanical cues is a critical driver of the glycolytic rate^43^.

ECM deposition in development and disease was shown to be regulated by CTGF^44^. *CTGF* null mice die shortly after birth due to respiratory defects^44^, but studies in developing *CTGF* mutant embryos have revealed an important role of CTGF in PC recruitment to the vasculature^18^. Noteworthy, reduced deposition of ColIV was also observed in CTGF mutants, thus indicating a role for CTGF in basement membrane formation^18^.

Similarly, in a mouse model targeting *CTGF* expression specifically in ECs^19^, impaired vascular cell growth and morphogenesis were detected in the postnatal retina. In these mice a key role for CTGF-YAP in retinal vascular development was reported^19^. In disease, in a mouse model of diabetic retinopathy, CTGF expression was required for thickening of the basal lamina of retinal capillaries, an instrumental step in the development of vision loss^21^ while in diabetic nephropathy, the increased CTGF expression was shown to inhibit BMP-7 signal transduction, reduce MMP activity and contribute to basement membrane thickening and albuminuria^45^.

In patients, targeting CTGF has mainly been associated with the development of fibrosis. CTGF was proposed as a surrogate marker of fibroproliferative disease for several pathologies^46^ while another study has reported preferential accumulation of the NH2-terminal CTGF fragment in proliferative diabetic retinopathy^47^. By far the most encouraging results were obtained in a phase 2 clinical trial using pamrevlumab (FG-3019), a recombinant human monoclonal antibody against CTGF, that was shown to be safe and effective in attenuating the progression of idiopathic pulmonary fibrosis^48^.

In our organoid model of human microvasculature, CTGF emerged as a paracrine regulator that can alleviate the vascular remodelling induced by the metabolic rewiring, by reinforcing the direct interaction of ECs and PCs and recovering the morphology of tight junctions. Our study highlights the presence of a CTGF-YAP feedforward loop in maintaining vascular integrity. We provide evidence that PFKFB3 is an upstream regulator of YAP, affecting its nuclear localisation and transcriptional activity.

In several different cell types (MCF10A, HepG2 and UOK262) blocking glucose metabolism, or shifting cellular metabolism away from glycolysis was shown to impair YAP/TAZ transcriptional activity and its ability to promote cell proliferation, cancer cell self-renewal and clonogenic potential in vitro^49^. In breast cancer, the lncRNA *BCAR4* was identified as a downstream target of YAP that controls cancer development, by reprogramming glucose metabolism through the transcription of two glycolysis activators, Hexokinase 2 (HK2) and PFKFB3. PFKFB3 inhibitors dramatically suppressed the YAP-dependent glycolysis, cell proliferation and tumorigenesis indicating the presence of a YAPBCAR4- glycolysis signalling axis^50^. In small-cell lung carcinoma cell lines PFKFB3 inhibition attenuated invasion/migration by downregulating YAP/TAZ signalling^51^.

YAP activity can be partially restored with supplementation with recombinant CTGF that can also recover the alterations in tight junction morphology induced by inhibition of glycolysis, effectively correcting the leaky thick junctions and bringing them to the levels observed in the control iPS-derived ECs, while enriching tight junctions for the migratory fingers junctions. This normalisation in tight junction morphology would have a beneficial effect on EC: PC direct interaction in BVOs. In our 3D model of the microvasculature, we observed a significant increase in PC coverage in microvessels and vessel density and length, indicating that CTGF can protect the microvasculature from structural remodelling triggered by metabolic rewiring.

Our study indicates that BVOs respond rapidly to metabolic changes. Organoids have emerged as promising tissue models for human biology and disease, which can bridge the gap between preclinical platforms and clinical trials. These self-organising 3D tissue units can recapitulate key aspects of in vivo organs, reflect human development and pathophysiology and capture human genetic heterogeneity^26^. In particular, organoids can offer unique insights into cell-to-cell and cell-to-ECM interactions in a human tissue setting. This is important as there are several examples in the literature where cells respond differently in 2D and 3D cultures^12,52,53^.

The prompt structural remodelling in response to metabolic rewiring in BVOs renders this model attractive for mechanistic studies and the identification of potential targets for novel therapeutic approaches. Importantly, this short timeframe of response is also useful and desirable for high throughput drug and small compound screening applications. The use of patient-derived iPS cells and BVOs that can capture the interpatient variation and predict drug responses more accurately could also facilitate the development of personalised treatment strategies.

## Methods

### Cell culture

HEK293T cells were purchased from LGC-ATCC (CRL-3216) and maintained in DMEM supplemented with 10% FBS. Human umbilical vein endothelial cells (HUVECs) were purchased from Promocell (C-12203) and cultured on gelatin-coated flasks in Endothelial Cell Growth Medium 2 (Promocell, C-22011) in a humidified incubator supplemented with 5% CO_2_. The cells were subcultured every three days at a ratio of 1:4. Cell culture images were taken using a Nikon Eclipse TS100 microscope and a Nikon DS-Fil camera.

### PFK15 treatment

The chemical selective 6-phosphofructo-2-kinase 3 (PFKFB3) inhibitor with the commercial name PFK15^25^ was purchased from Selleckchem (S7289). The inhibitor can be stored as a stock solution of 50 mM in dimethyl sulfoxide (DMSO) at −80 °C. Once thawed, aliquots may be kept at 4 °C for 2 weeks. Unless otherwise stated, BVOs or iPS-ECs were washed twice and then incubated in EBM2 with no supplement (BVOs for 2 h, iPS-ECs for 1 h). Subsequently, they were treated for the indicated time with PFK15 (2.5 µM) or DMSO in EBM2 media in the absence of supplements.

### AZ PFKFB3 67 treatment

AZ PFKFB3 67 (referred to herein as AZ67), a structure-based chemical inhibitor of PFKFB3 was purchased from TOCRIS (5742). The inhibitor was resuspended in DMSO to 50 mM concentration and stored at −20 °C. Unless otherwise stated, BVOs were washed twice and then incubated in EBM2 with no supplement (BVOs for 24 h). Subsequently, they were treated for the indicated time with AZ67 (0.5 µM or 1 µM) or DMSO in EBM2 media in the absence of supplements.

### Generation of blood vessel organoids

BVOs were generated from iPS cells using the protocol established by Wimmer et al.^22^. The KOLF2 human iPS cell line was obtained from the Wellcome Trust Sanger Institute. hiPSCs were plated on Matrigel-coated (ATCC, ACS-3035) 6-well plates and cultured using the StemMACS iPS Brew-XF system (Miltenyi Biotech, 130-104-368). For differentiation to BVOs, 2.5 × 10^5^ iPSCs per well were seeded into Ultra-low adherent 6-well plates (Appleton Woods, 3471) in Aggregation Media to form cell aggregates (diameter average 50–150 µm) for approximately one day. Aggregates were collected by gravitation (Day 0) and resuspended into the mesodermal induction media (N2B27 Media supplemented with 12 μM CHIR 99021 (Tocris Bioscience, 4423) and 30 ng/ml BMP4 (ThermoFisher, PHC9534)^22^. On day 3, the aggregates were collected by gravitation and plated in N2B27 media supplemented with 100 ng/ml VEGFA (Peprotech, 100–20) and 2 μM Forskolin (R&D system, 1099) for vascular lineage promotion. On day 5, the aggregates were embedded in a substrate of Collagen I-Matrigel (Advanced BioMatrix, 5005) (ratio 4:1) with StemPro-34 SFM (Thermo Fisher, 10639011) supplemented with 15% FBS (Thermo, 10500064), 100 ng/ml VEGFA and 100 ng/ml FGF2 (Miltenyi Biotech, 130-093-841) to induce vascular network formation^22^. The sprouting appeared within two days in all preparations. Fresh media was provided after three days and then every other day. Vascular networks (VNs) were established between days 10 to 12 and extracted from the Matrigel to a 96-well ultra-low-attachment plate (2BScientific, MS-9096UZ) where they self-assembled to BVOs. BVOs were kept in culture for up to 40 days. For hypoxia treatment, BVOs were exposed to 1%O_2_ for 24 h in a ProOX Model C21 incubator (BioSpherix Ltd).

Vessel density and length were quantified using Vessel Analysis Plugin on ImageJ (Fiji, version 1.53t) as a percentage (%) of the area analysed. ColIV deposition was quantified using ImageJ (Fiji, version 1.53t). Stack images were resliced along a vertical axis and cross-sections were used for quantification.

### Genome editing using CRISPR-Cas9

A single guide RNA targeting *PFKFB3* was designed using the CRISPR Finder online tool (Wellcome Sanger Institute Genome Editing) and cloned into a mammalian expression vector encoding Cas9 from *S. pyogenes* and a puromycin resistance gene (Addgene, 62988) using the Golden gate reaction^54^. The following primers were used for PFKFB3 guide RNA cloning: PFKFB3 F: CACCGGCCCACCATGACGATGACGG

PFKFB3 R: AAACCCGTCATCGTCATGGTGGGCC

The sgRNA vector was confirmed by Sanger sequencing and transfected into iPS cells using Lipofectamine 3000. In brief, 5 µg of plasmid were transfected into iPS cells seeded on matrigel-coated six-well plates in StemMACS iPS Brew-XF media (Miltenyi Biotech, 130-104-368) supplemented with 10 μM Y-27632 for 24 h. The following day, the transfection complexes were removed and fresh StemMACS iPS Brew-XF media supplemented with 10 μM Y-27632 was added to the cells. Puromycin selection (0.2 µg/ml) was performed for 48 h and single cells were cultured for an additional ten days. Single colonies representing isogenic mutant lines were selected and expanded. Knockout cell lines were verified by Sanger sequencing and western blot analysis. Two independent knockout clones (P206, P208) were used for this study.

### Differentiation of iPS-EC

The method used was adapted from previously published protocols^23^. iPS cells were seeded on Corning Matrigel Growth Factor Reduced (GFR) Basement Membrane Matrix (SLS, 356231) at a density of 1.6 × 10^5^ cells per 6 well in StemMACS iPS-Brew XF (Milteney Biotech, 130-104-368) supplemented with 10 μM Y-27632 (ATCC ACS-3030) for 24 h. The following day (day 1), the medium was changed to N2B27 medium, a 1:1 ratio of Neurobasal medium (Thermo, 21103049) and DMEM/F12 (Gibco, 11330-032), with N2 (Thermo, 17502048), B27 (Thermo, 12587010), Glutamax (Thermo, 350050061) and freshly supplemented with 8 μM of CHIR (Sigma, SML1046) and 25 ng/ml of BMP4 (ThermoFisher, PHC9534). After 72 h (day 4), the medium was replaced with StemPro-34 SFM (Gibco, 10639011) supplemented with 200 ng/ml human VEGFA (Peprotech, 100-20) and 2 μM Forskolin (R&D system, 1099). On day 6, cells were selected by Magnetic Activated Cell Sorting (MACS) for iPS-EC expressing CD144 using Microbeads Kit (Miltenyi Biotec, 130-097-857). Positive cells were seeded on mouse Collagen IV (BioTechne, 3410-010-02) coated plate in EGM2-MV medium (Promocell, C-22011) supplemented with 20% FBS (Thermo, 10500064) and 50 ng/ml VEGFA. Cells were used for experiments up to 3 passages.

### Metabolic analyses

To assess the metabolic fate of glucose, iPS-ECs were cultured with 5 mM U-^13^C_6_-glucose (Cambridge Isotope Laboratories, CLM-1396-1) in DMEM without glucose and pyruvate (Thermo Fisher Scientific, 12307263) supplemented with 1% dialysed FBS, 1% penicillin-streptomycin and 1% l-glutamine for 7 h, as pilot studies indicated that cells reached isotopic steady-state by this time. For BVOs, a 3 h incubation with 5 mM U-^13^C_6_-glucose (Cambridge Isotope Laboratories) in DMEM without glucose and pyruvate (Thermo Fisher Scientific) supplemented with 1% penicillin-streptomycin and 1% l-glutamine was performed. The medium was then removed and 80:20 methanol: water (−80 °C, extraction solvent) was added to cells for 15 min at −80 °C for metabolic quenching. Cells were scraped in the extraction solvent and cell lysates were pipetted into Eppendorf tubes followed by a sonication step. Then, samples were centrifuged at 16,000 × *g* for 10 min at 4 °C to pellet debris.

The supernatant was transferred to a new tube and dried using a SpeedVac (Thermo Fisher Scientific, Savant, SPD131DDA). The dried pellet was resuspended in 50 µl chloroform /100 µl methanol/100 µl H_2_O. The top, polar fraction was further dried and stored at −80 °C until LC-MS analysis. All metabolite analyses were performed on three biological replicates.

### LC-MS analysis

Liquid chromatography tandem mass spectrometry (LC-MS/MS) was employed^24^. The dried polar metabolite fractions were reconstituted in acetonitrile/water (v/v 3/2), vortexed and centrifuged at 16,000 × *g* for 3 min before analysis on a 1290 Infinity II ultrahigh performance liquid chromatography (UHPLC) system coupled to a 6546-quadrupole time-of-flight (Q-TOF) mass spectrometer (Agilent Technologies). Samples were separated on a Poroshell 120 HILIC-Z column (100 × 2.1 mm, 2.7 μm, Agilent) attached to a guard column (5 × 2.1 mm, 2.7 µm) and analysed in negative ionisation mode using water with 10 mM ammonium acetate (solvent A) and acetonitrile with 10 mM ammonium acetate (solvent B), both solvents containing 5 µM Infinity Lab deactivator additive (Agilent Technologies). The elution gradient used was as follows: isocratic step at 95% B for 2 min, 95 to 65% B in 12 min, maintained at 65% B for 3 min, then returned to initial conditions over 1 min, and then the column was equilibrated at initial conditions for 8 min. The flow rate was 0.25 ml/min; the injection volume was 5 μl, and the column oven was maintained at 30 °C. Feature annotation and metabolite identification were based on accurate mass and standard retention times with a tolerance of ±5 ppm and ±0.5 min, respectively, and performed with MassHunter Profinder (version 10.0.2, Agilent Technologies) using our in-house curated metabolite library based on metabolite standards (Sigma-Aldrich). ^13^C label incorporation levels were normalised to the natural occurrence of ^13^C isotopes and are represented as corrected abundance percentages. Samples were run in one batch and injected into technical duplicates.

### Seahorse assays

Extracellular flux analyses studies were performed using a Seahorse XF^24^ analyzer (Agilent) according to the manufacturer’s protocol. Cells were plated on substrate-coated Seahorse XFe24 culture plates at 3 × 10^4^ cells/well with the respective substrate in complete media. For mitochondrial stress test, the following day the media were changed to unbuffered DMEM (Sigma-Aldrich, D5030) supplemented with either 10 mM Glucose or 1 mM Sodium pyruvate or 2 mM Glutamine, pH 7.4, at 37 °C, 1 h before assay. Basal oxygen consumption rate (OCR), ATP-linked OCR and maximal respiration were determined by the sequential administration of 1 µM oligomycin (Sigma-Aldrich, 75351), 2 µM Carbonyl cyanide 4-(trifluoromethoxy)phenylhydrazone (FCCP) (Sigma-Aldrich, C2920), and 1 µM antimycin A (Sigma-Aldrich, A8674) and 2 µM rotenone (Sigma-Aldrich, R8875). For fatty acid oxidation (FAO) experiments, 1 mM BSA-conjugated palmitate was added in each well just before starting the experiment.

For glycolysis, on the day of the experiment the media was changed for unbuffered DMEM (Sigma-Aldrich, D5030) pH 7.4, at 37 °C, 1 h before assay and the extracellular acidification rate (ECAR) was determined at baseline, and after the sequential injection of 10 mM Glucose, 1 µM oligomycin (Sigma-Aldrich, 75351) and 50 mM d-deoxy d-Glucose (Sigma-Aldrich, D6134). At the end of the experiment cells were fixed using 4% PFA for 15 min and treated with 5 µM DRAQ5^TM^ (Thermo Scientific, 62251) for 20 min. Cell nuclei were imaged and analysed using Li-cor Odyssey imager and quantification was used as an index of cell number for data normalisation.

### Immunohistochemistry

BVOs were rinsed twice in PBS and then fixed in 4% paraformaldehyde (PFA) for 1 h at room temperature (RT) and washed twice in PBS. BVOs were dehydrated in 20% sucrose at 4 °C overnight, then embedded in Gelatin solution and stored at −20 °C for cryosectioning. The frozen BVOs were sectioned using NX70 Cryostat (Thermo Scientific) to 20 and/or 70-μm thickness. Frozen sections were then washed in PBS for 5 min and then permeabilized and blocked in Blocking buffer for 2 h at RT. Primary antibodies were diluted in Blocking buffer and incubated overnight in a cold room. Antibodies used and dilution factors’ information are available in Supplementary Table S1. On the following day, sections were washed three times in PBS with 0.1% TritonX-100 (PBS-T) and then incubated with labelled secondary antibodies for 2 h at RT. After two washes in PBS-T, the sections were counterstained with DAPI and mounted with Fluoromount-G mounting media (Thermo, 00-4958-02).

For immunostaining intact BVOs and VNs were rinsed in PBS then fixed in 4% PFA for 30 min at RT and washed twice in PBS. The fixed VNs were stored in PBS at 4 °C for up to a month. Stained BVOs were mounted into iSpacer (0.5 mm deep imaging Spacer, SunJin Lab). Samples were viewed and imaged using the Spinning Disk Confocal System (Nikon) and the Operetta CLS High-Content Analysis System (PerkinElmer) at a 20x or 40x magnification.

### Quantitative reverse transcription PCR

RNA was isolated using the PureLink RNA Mini kit (ThermoFisher Scientific, 12183018 A) according to the manufacturer’s recommendation and reverse-transcribed into cDNA using High-Capacity cDNA Reverse Transcription Kit (ThermoFisher, 4368813). The reactions were conducted using SYBR green master mix (ThermoFisher, 4472920) on the ViiA 7 real-time PCR system (ThermoFisher) at 95 °C for 10 min, followed by 40 cycles of 95 °C for 15 sec and 60 °C for 1 min. *Beta-actin* was used as a normalisation control. The following primers were used: CCN2 (*CTGF*) F CCGCACAAGGGCCTATTCT, CCN2 R GGTACACCGTACCACCGAAG, MMP9 F CCTGGGCAGATTCCAAACCT, MMP9 R CAAAGGCGTCGTCAATCACC, B-actin F CGTCTTCCCCTCCATCGTG, B-actin R CTCGATGGGGTACTTCAGGG.

### Flow cytometric analysis

BVOs (*n* = 7 per group) were mechanically dissociated using a scalpel and then incubated in Dissociation solution (1.7 mg Dispase (Gibco, 17105-041), 0.2 mg Liberase (Roche, 05401135001) and 0.1 mg DNase (Stem Cell Technologies, 07900) per ml) in PBS for 20 min at 37 °C. BVO solutions were passed up to 10 times through 21 g needles. Approximately 50,000 single cells were resuspended in 100 μl of FACS buffer (PBS containing 1% FBS) and stained with fluorescence-conjugated antibodies for 30 min at 4 °C in the dark. Information for the antibodies used and dilution factors is listed in Supplementary Table S2. The cells were washed in PBS and resuspended in 1% PFA in PBS. Data were acquired the following day using a LSRFortessa Flow Cytometer analyzer (Becton, Dickinsin and Company) and analysed using FlowJo software (Becton, Dickinson and Company).

### Western blot

Whole protein lysates were isolated from iPS cells and iPS-ECs using the 1x Cell Lysis buffer (Cell Signalling, 9803) supplemented with protease inhibitors (Roche, 11697498001). Cells were washed using PBS and harvested at the same passage and confluency (80%) to minimise differences between samples. Proteins were quantified using the BCA method (ThermoFisher Scientific, 23227). A total of 16 μg of proteins were loaded and separated on 4–12% polyacrylamide-SDS gels under reducing conditions (Invitrogen, NP0322) and transferred to nitrocellulose membranes (Amersham, 10600003).

Membranes were blocked in 0.1% Tween20 TBS (TBS-T) and 5% dry non-fat milk for 1 h at RT and incubated with the different primary antibodies overnight at 4 °C. The following day, membranes were washed three times for 10 min with TBS-T and incubated for 1.5 h at RT with the respective horseradish peroxidase (HRP)-conjugated secondary antibodies (anti-rabbit, 211-032-171, and anti-mouse, 115-035-174, Stratech with 1:4000 dilution). The ECL detection system (GE Health, GERPN2232) was used. The antibodies used and the dilution factors’ information are listed in Supplementary Table S3.

### Nuclear/cytoplasmic fractionation

Nuclear and cytoplasmic fractionation of iPS-ECs following PFK15 treatment was performed^55^. IPS-ECs were seeded on ColIV in 6-well plates (2 × 10^5^ cells per well) and the following day treated with PFK15 as above for 3 h. The cells were subsequently washed with ice-cold PBS and harvested in 80 μl of hypotonic buffer (50 mM Tris pH 7.5, 10 mM NaCl, 3 mM MgCl_2_, 10% glycerol) supplemented with protease inhibitors (complete tablets, Mini EDTA-free, Roche) and phosphatase inhibitors (Phosphatase Inhibitor Cocktail 3 Sigma P0044, 2 mM orthovanadate, 1 mM NaF). After 1 min, cells were scraped and 0.1% NP40 was added to the cells, for a 5 min incubation on ice. The cells were centrifuged at 1500 × g for 5 min at 4 °C and the supernatant representing the cytosolic fractions were then recovered. Pellets were resuspended in 70 μl IPLS buffer (50 mM Tris pH 7.5, 120 mM NaCl, 1 mM EDTA, 0.5% NP40, supplemented with inhibitors as above) and sonicated with 3 pulses of 10 min each (30 sec on/ 30 sec off) on ice in a Bioruptor Plus (Biosense). To obtain the nuclear fraction, the lysates were centrifuged at 21,300 × *g* for 20 min at 4 °C and the supernatant was recovered as the nuclear fraction. Lysates (10 μg) were loaded for protein separation and subsequent blotting onto a PVDF membrane (Amersham Hybond PVDF, GE Healthcare).

### Zymography

The conditioned media was harvested from BVOs following 24 h of treatment and cell debris was removed by centrifugation at 1500 × *g* for 10 min. The supernatant was transferred into a new tube and stored at −80 °C. Samples were mixed with non-reducing Tris-Glycine SDS sample buffer (ThermoFisher, LC2675) and loaded to a Novex® 10% zymogram gelatin gel (ThermoFisher, ZY00102BOX). After electrophoresis, the gel was renatured and developed at 37 °C overnight according to the manufacturer’s recommendations. The gel was stained with Simply blue Safestain (ThermoFisher, LC6060). Data were quantified using the ImageJ (Fiji, version 1.53t) and normalised to total protein content, as measured by total protein spectra.

### Luciferase reporter assays

Luciferase assays were performed in HEK293T cells using the YAP/TAZ reporter 8xGTIIC-lux (Addgene, 34615). *Renilla*-Luc (0.05 µg/well) was included as an internal control. Cells were plated in 12 well plates (10^5^ cells/ 12 well) and the following day transfected using Lipofectamine 2000 (ThermoFisher Scientific, 11668-027) transfection reagent. The PFK15 and recombinant CTGF as indicated per condition were supplemented to the wells two hours after the initiation of transfection. Luciferase activity was detected 24 h after transfection and normalised to Renilla luciferase activity using the Dual-Glo Luciferase system (Promega, E2920). The relative luciferase unit was defined as the ratio of Firefly versus Renilla. A total of three independent transfections were performed in quadruplicates.

### In vitro tube formation assay

For in vitro tube formation, 24-well plates were coated with 200 μl/well of Matrigel Matrix (Corning, 354234). The plates were incubated at 37 °C for 30 min. Subsequently, 6 × 10^4^ iPS-ECs were plated in each well and incubated for 4 h, at 37 °C in a humidified incubator supplemented with 5% CO_2_. Vascular network images were taken using a Nikon Eclipse TS100 microscope and a Nikon DS-Fil camera.

### Proteomic Analysis of the BVO secretome

Secreted proteins were concentrated using 3kD MWCO spin filters (Amicon) at 20,000 × *g*, 4 °C. The protein samples were denatured by 6 M urea, 2 M thiourea and reduced by 10 mM DTT at 37 °C for 1 h, alkylated by 50 mM iodoacetamide at RT in the dark for 1 h, and washed with 0.1 M triethylammonium bicarbonate (TEAB, pH=8.5) for 3 times in the spin filter. The proteins were digested by Trypsin/LysC Mix (protein: enzyme = 25:1, Millipore) at 37 °C overnight. Peptides were acidified by 1% trifluoroacetic acid (TFA) and C18 cleanup was performed using Bravo AssayMAP robot with C18 cartridges (Agilent) following the manufacturer’s instruction. Eluted peptides were SpeedVac dried and resuspended in 2% acetonitrile (ACN), 0.05% the TFA in LC-MS grade H_2_O.

Peptide samples were injected and separated by an UltiMate3000 RSLCnano system (EASY-Spray C18 reversed-phase column, 75 µm x 50 cm, 2 µm, Thermo Fisher Scientific) using the following LC gradient: 0–1 min: 1% B; 1–6 min: 1–6% B; 6–40 min: 6–18% B; 40–70 min: 18–35% B; 70-80 min: 35–45% B; 80–81 min: 45–99% B; 81–89.8 min: 99% B; 90–120 min: 1% B (A = 0.1% formic acid in H_2_O, B = 80% ACN, 0.1% formic acid in H_2_O). The separated peptides were directly injected into an Orbitrap Q Exactive HF Mass Spectrometer (Thermo Fisher Scientific). Full MS spectra were collected using Orbitrap with a scan range of 375–1500 m/z and a resolution of 60,000. The most abundant 15 ions from the full MS scan were selected for data-dependant MS2 with HCD fragmentation and acquired using Orbitrap with a resolution of 15,000 and isolation windows 2 *m*/*z*. Dynamic exclusion of 40 sec and lock mass of 445.12003 m/z were used.

RAW data were analysed using Proteome Discoverer (version 2.4, Thermo Fisher Scientific) with MASCOT algorithm (version 2.6.0, Matrix Science). The following parameters were used: UniProt/SwissProt human and bovine protein database (version 2021_01, 26410 protein entries) were used; trypsin was used as an enzyme with maximum 2 missed cleavages allowed; carbamidomethylation on cysteines was selected as static modification and oxidation on methionine, lysine and proline was selected as dynamic modifications; precursor ion mass tolerance was set at 10 ppm, and fragment mass tolerance was set at 20 milli-mass unit (mmu). Protein identification FDR confidence was set to High and the minimum number of peptides per protein was 2. The precursor peak area was used for quantification and exported for further statistical analysis.

### Bioinformatic analysis

For both proteomics and metabolomics dataset comparisons we applied an established omics-based preprocessing and statistical analysis pipeline (https://github.com/konstantinostheofilatos/Vascular_Proteomics-Statistical_Analysis). Datasets filtered to keep only proteins/metabolites with less than 30% missing values. All remaining missing values were imputed with the KNN-Impute method with k equal to 2. The relative quantities of the proteins were scaled using log2 transformation.

The limma package^56^ has been used to compare different phenotypes using the Ebayes algorithm and correcting for selected covariates. The nominal *p*-values were adjusted for multiple testing using Benjamini-Hochberg method^57^ but because of the limited sample size, a combination of thresholds of 0.05 on the nominal *p*-values and 0.25 for the absolute log2 fold change were used to infer statistically significant changes. Beanplots, Volcano Plots and heatmaps plots were constructed using the Beanplots^58^,Ggplot2^59^ and Corrplot^60^ packages of the R programming environment^61^, using R version 4.2.1.

Network visualizations were conducted using Cytoscape tool^62^ with protein-protein interaction networks being reconstructed from String web tool^63^. Pathway and functional enrichment analysis were conducted using DAVID tool^64^. This analysis included pathway terms from Reactome Pathway Database^65^, KEGG^66^ and functional terms from Gene Ontology^67^. Significantly enriched terms were inferred with a Benjamini-Hochberg adjusted p-value threshold of 0.05. Transcriptional factor enrichment analysis was performed using the ChEA3 tool^68^ using the ENCODE ChIP-sequencing data^69^.

### Statistics

Biological replicates were performed in all the experiments with *n* = 3 independent preparations. Data are expressed as the mean ± SD or mean ± SEM as indicated and were analysed using GraphPad Prism 9 software with a two-tailed Student’s *t* test for two groups or ANOVA for more than two groups. A value of *p < 0.05, **p < 0.01, ***p < 0.001, ****p < 0.0001 was considered significant.

### Reporting summary

Further information on research design is available in the Nature Portfolio Reporting Summary linked to this article.

### Supplementary information

Supplementary Information

Description of Additional Supplementary Files

Supplementary Dataset 1

Supplementary Dataset 2

Supplementary Dataset 3

Supplementary Dataset 4

Supplementary Movie 1

Supplementary Movie 2

Reporting Summary

### Source data

Source Data

## Data Availability

All data generated or analysed during this study are included in this manuscript and in its supplementary information files. Source data are provided with this paper. The mass-spectrometry proteomics data generated and analysed during the current study have been deposited to the ProteomeXchange Consortium via the PRIDE partner repository with the dataset identifier PXD041780. Source data are provided with this paper.
