# Supplementary material for: Human blood vessel organoids reveal a critical role for CTGF in maintaining microvascular integrity
Source: Nat Commun. 2023 Sep 9;14:5552. doi: 10.1038/s41467-023-41326-2 (PMC10492781; doi:10.1038/s41467-023-41326-2)

Supplementary Material

Human blood vessel organoids reveal a critical role for CTGF in maintaining microvascular integrity

Sara G Romeo[1], Ilaria Secco[1], Edoardo Schneider[1], Christina M Reumiller[1], Celio XC Santos[1], Anna Zoccarato[1], Vishal Musale[1], Aman Pooni[1], Xiaoke Yin[1], Konstantinos Theofilatos[1], Silvia Cellone Trevelin[1], Lingfang Zeng[1], Giovanni E Mann[1], Varun Pathak[2], Kevin Harkin[2], Alan W Stitt[2], Reinhold J Medina[2], Andriana Margariti[2], Manuel Mayr[1], Ajay M Shah[1], Mauro Giacca[1] and Anna Zampetaki[1*]

[1]King's College London British Heart Foundation Centre, School of Cardiovascular & Metabolic Medicine and Sciences, London, United Kingdom; [2] The Wellcome-Wolfson Institute for Experimental Medicine, Queen's University Belfast, Belfast, United Kingdom.

***Corresponding author:** Dr Anna Zampetaki, School of Cardiovascular & Metabolic Medicine and Sciences, James Black Centre, 125 Coldharbour Lane, London SE5 9NU, UK. Email: anna.zampetaki@kcl.ac.uk

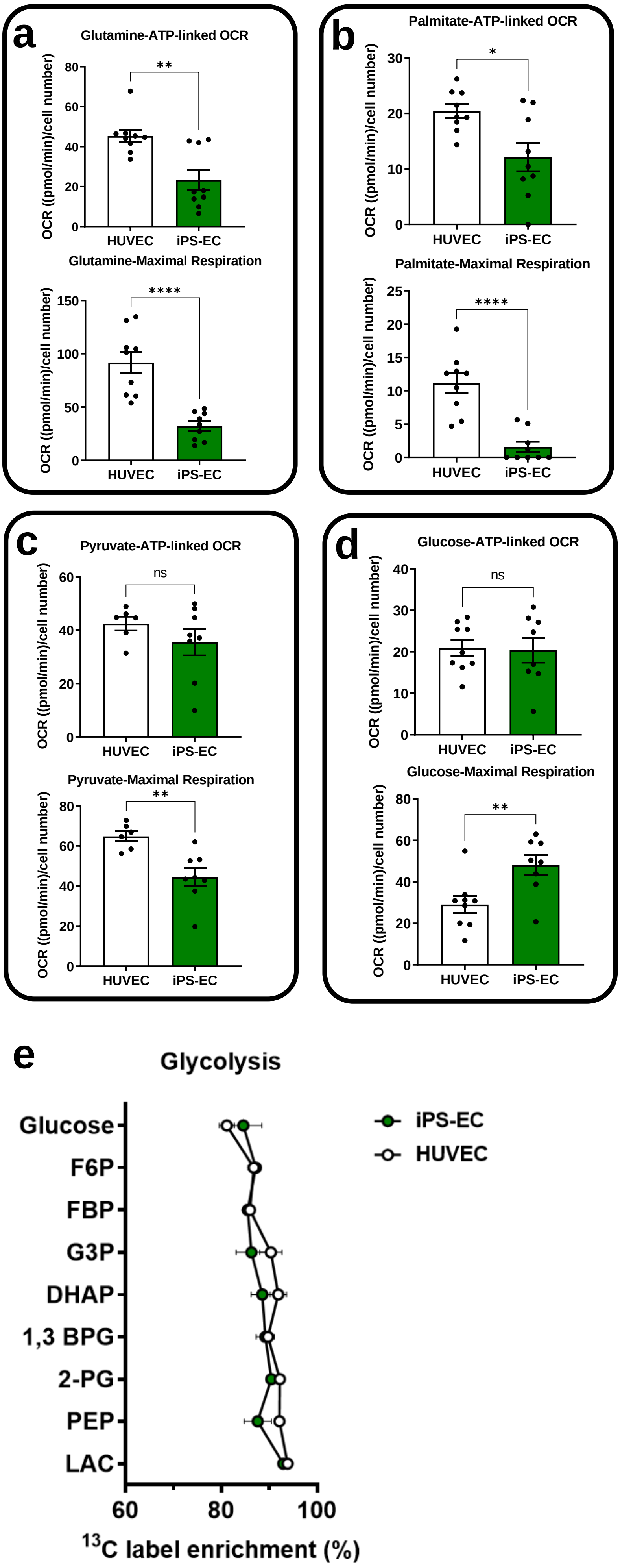

Supplementary Figure 1. Substrate utilisation in iPS-ECs and HUVEC as assessed by Seahorse assays. (a) glutamine, **(b)** palmitate, **(c)** pyruvate, **(d)** glucose, ATP-linked OCR and maximum respiration are shown. Three independent lines were assessed in n=3 wells per assay. Values are presented as mean ± SEM; P values were calculated using a two-tailed Student's t-test. (**a**: **p=0.0019; ****p=0.000064; **b**: *p=0.0101; ****p=0.000038; **c**: **p=0.0037; **d**: **p=0.0083). ns= not significant. OCR (Oxygen Consumption Rate). **(e)** ^{13}C label incorporation into glycolytic intermediates in HUVEC and iPS-EC cell lines at baseline after 7h of incubation with $^{13}C_6$-glucose. Data represents mean ±SEM, n=3, independent experiments for iPS-ECs and n=4, independent experiments for HUVEC statistical significance was assessed by a two-way ANOVA with Holm-Sidak post-hoc test, Abbreviations: F6P (fructose 6-phosphate). FBP (fructose 1,6-bisphosphate), G3P (Glyceraldehyde 3-phosphate), DHAP (Dihydroxyacetone phosphate), 1,3-BPG (1,3-bisphosphoglycerate), 2-PG (2-phosphoglycerate), PEP (phosphoenolpyruvate), LAC (Lactate).

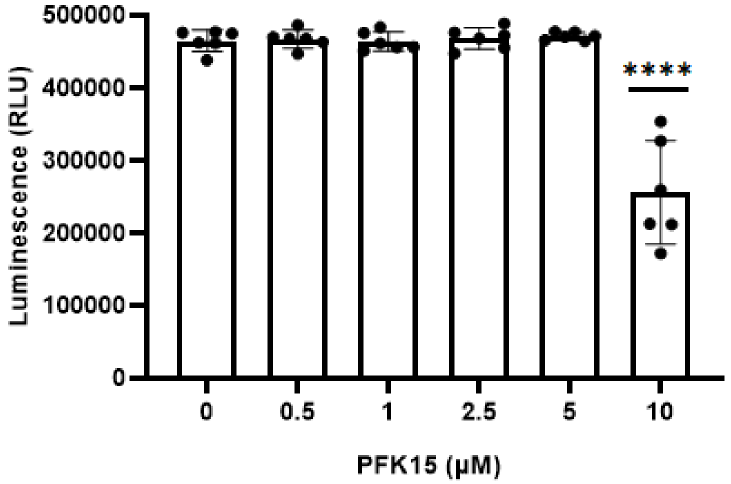

Supplementary Figure 2. Survival of iPS-ECs treated with various concentrations of PFK15 for 24h. Values are presented as mean ± SD; P values were calculated using one-way ANOVA followed by Tukey's multiple comparisons tests (****p=0.000000000000420) n=2 independent experiments.

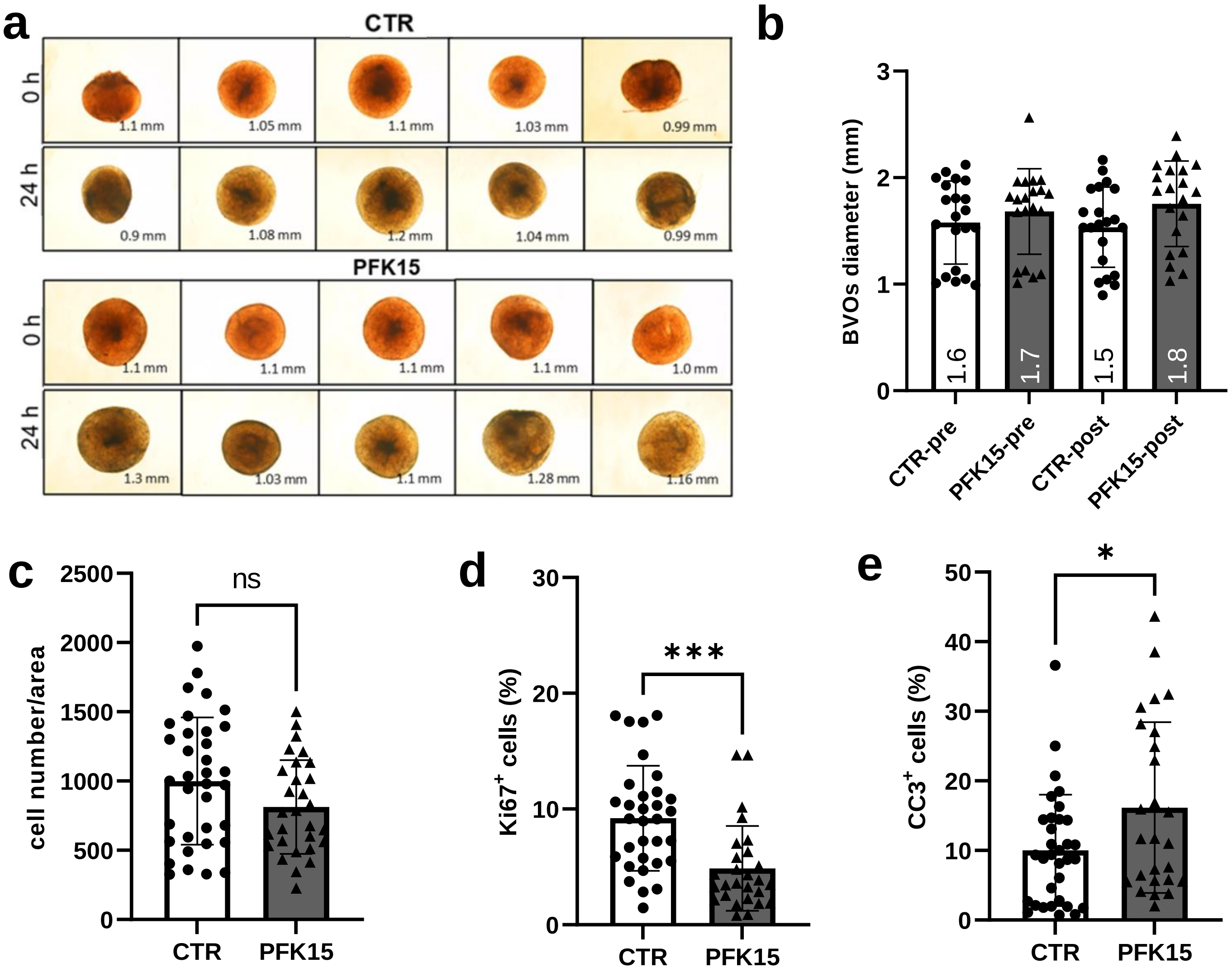

Supplementary Figure 3. The effect of PFK15 on BVO diameter and cellular composition. (a) Bright-field images of BVOs treated with DMSO (CTR) or PFK15 (2.5 μM) for 24h and **(b)** quantification of BVO diameter pre- and post-treatment n=20 BVOs per group. **(c)** Total number of cells per area, **(d)** percentage of proliferating cells, and **(e)** percentage of cleaved caspase 3 (CC3) positive cells in n=15 BVOs per group from 5 separate preparations. One-two sections per BVO were assessed. Values are presented as mean ± SD; P values were calculated using a two-tailed Student's t-test. (**d**: ***p=0.0002; **e**: *p=0.0237). ns= not significant.

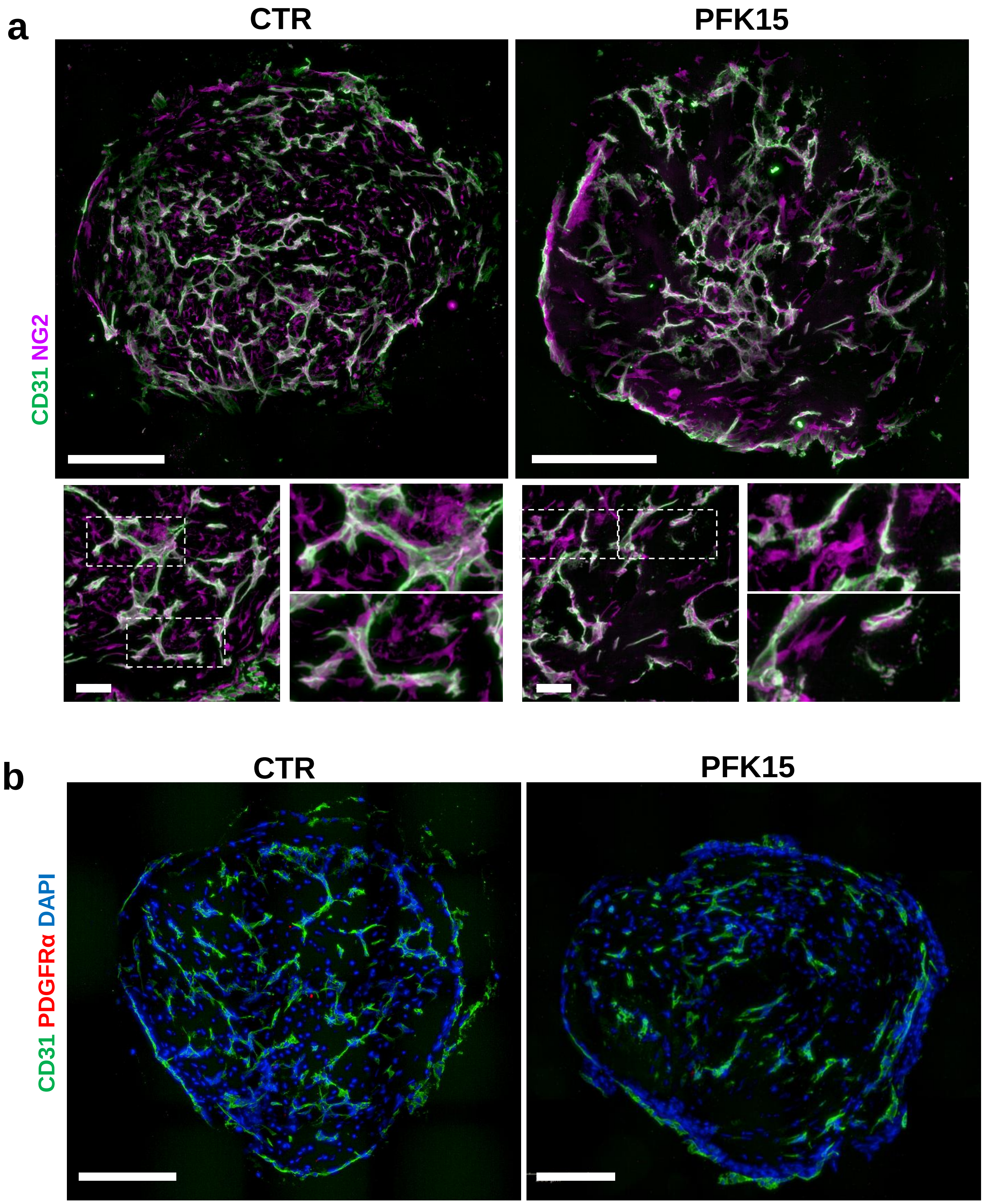

Supplementary Figure 4. NG2 expression in BVOs following PFK15 treatment. Phenotypic characterization of **(a)** NG2 (magenta) and **(b)** PDGFRα (red) expression in BVO sections using immunofluorescence confocal microscopy. CD31⁺ ECs are shown in green. DAPI is shown in blue. N=5, representative images of 5 independent experiments. Bar scales 200 μm and 50 μm.

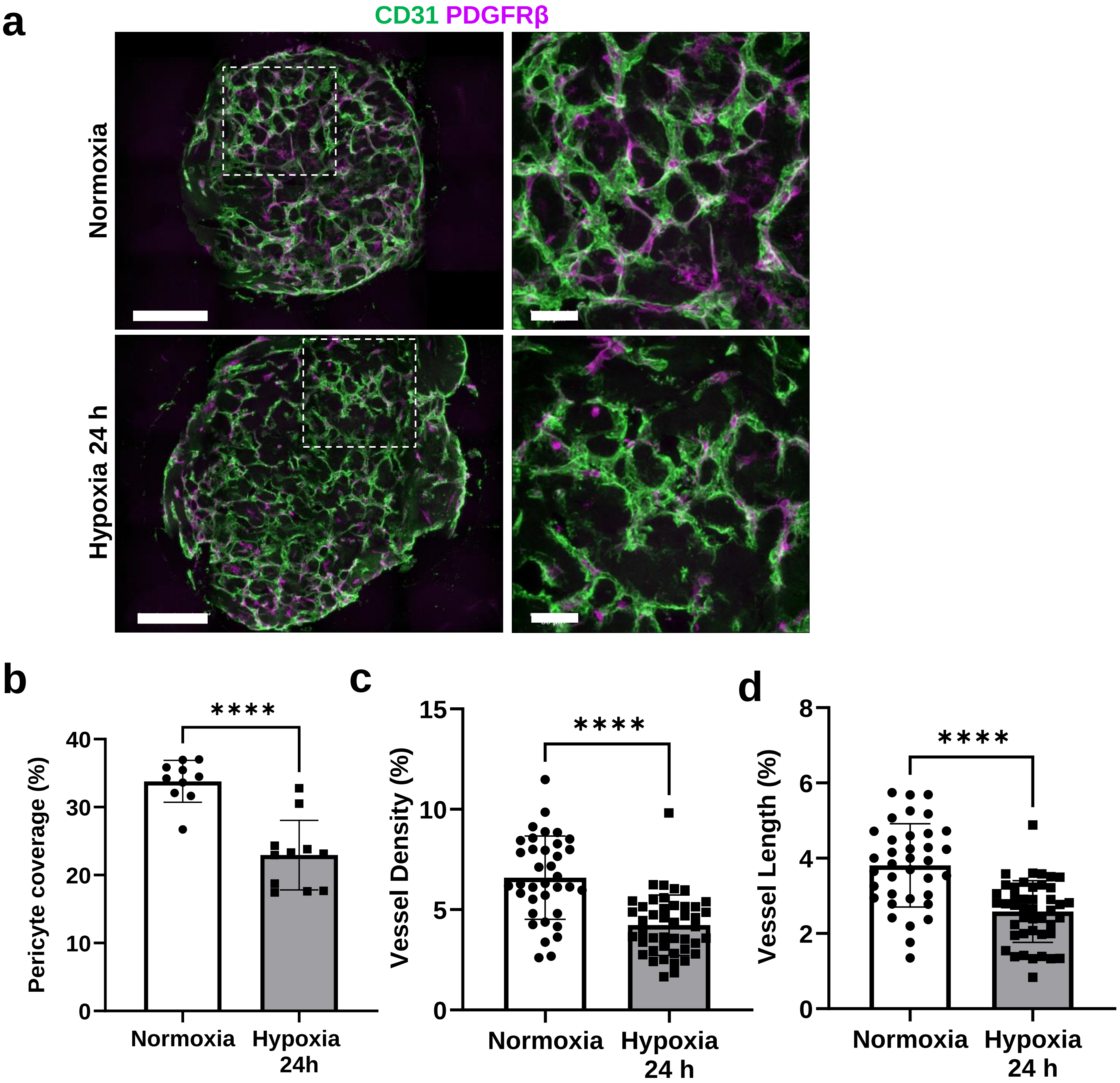

Supplementary Figure 5. BVOs' structure under hypoxia. (a) Immunofluorescence confocal imaging showing $CD31^{+}$ ECs (green) in vascular networks covered by pericytes ($PDGFR\beta^{+}$, magenta) in sections from BVOs under normoxia or hypoxia condition for 24hs. **(b)** pericyte coverage, n=6 BVOs per group, from 3 separate BVO preparations. One or two sections per BVO were assessed. **(c)** quantification of vessel density and **(d)** vessel length. N=6 BVOs per group, from 3 separate BVO preparations and 4 different areas per 10x images have been used for quantification. One or two sections per BVO were assessed. Data are shown as mean ± SD a two-tailed Student's t-test. (**b**: ****p=0.000013; **c**: : ****p=0.000000095; **d**: ****p=0.000000199). Bar scales 200 μm (left) and 50 μm (right).

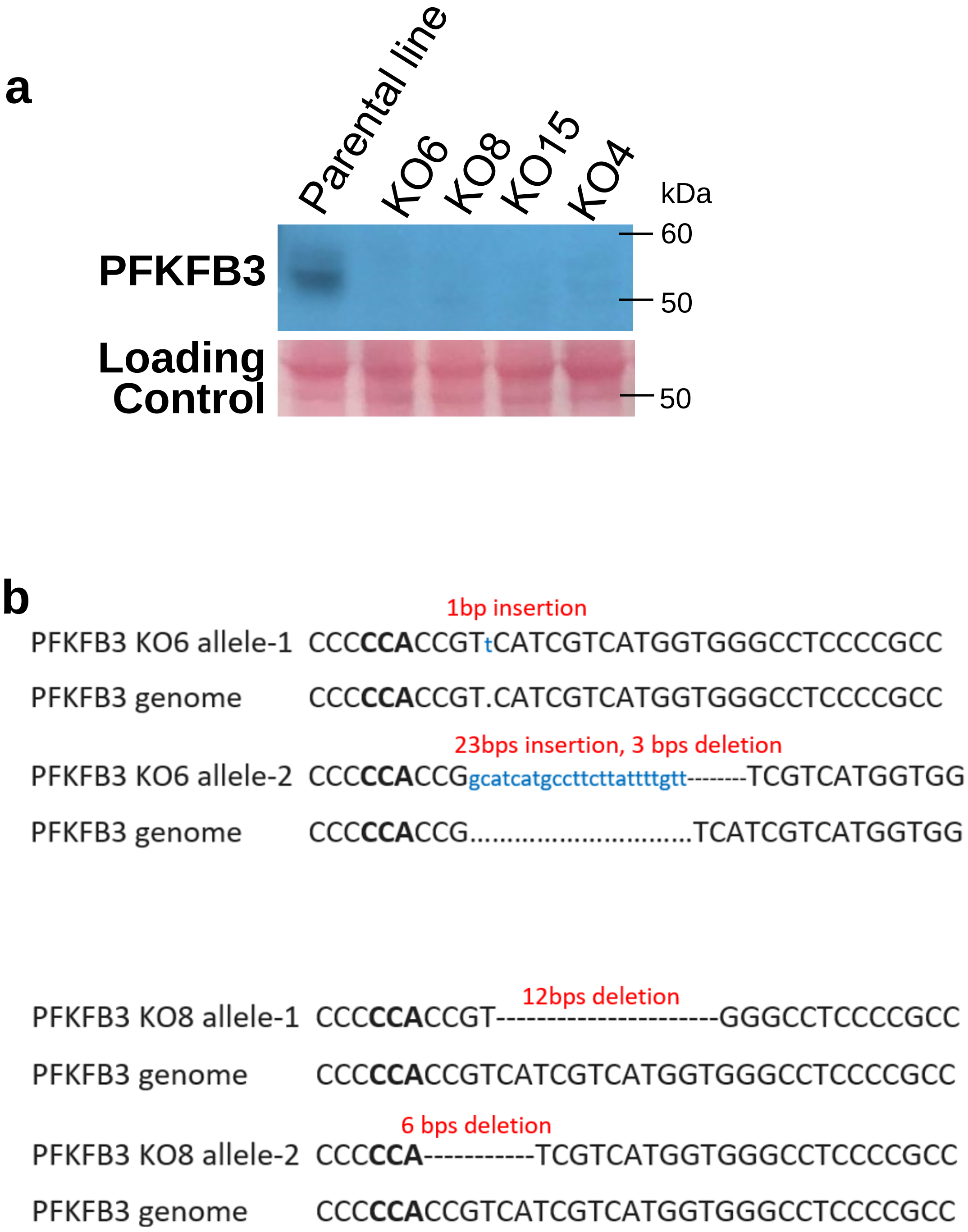

Supplementary Figure 6. Generation of *PFKFB3* knockout iPS lines. (a) CRISPR/Cas9 genome editing was used to generate *PFKFB3* knockout iPS lines that lacked the expression of PFKFB3, as determined by western blot analysis. **(b)** Sanger sequencing of genomic DNA revealed the presence of indels in KO6 clone (referred to herein as P206) and deletions in KO8 clone (referred to herein as P208). The PAM sequence is highlighted in bold, insertions are shown in blue and deletions are noted in red.

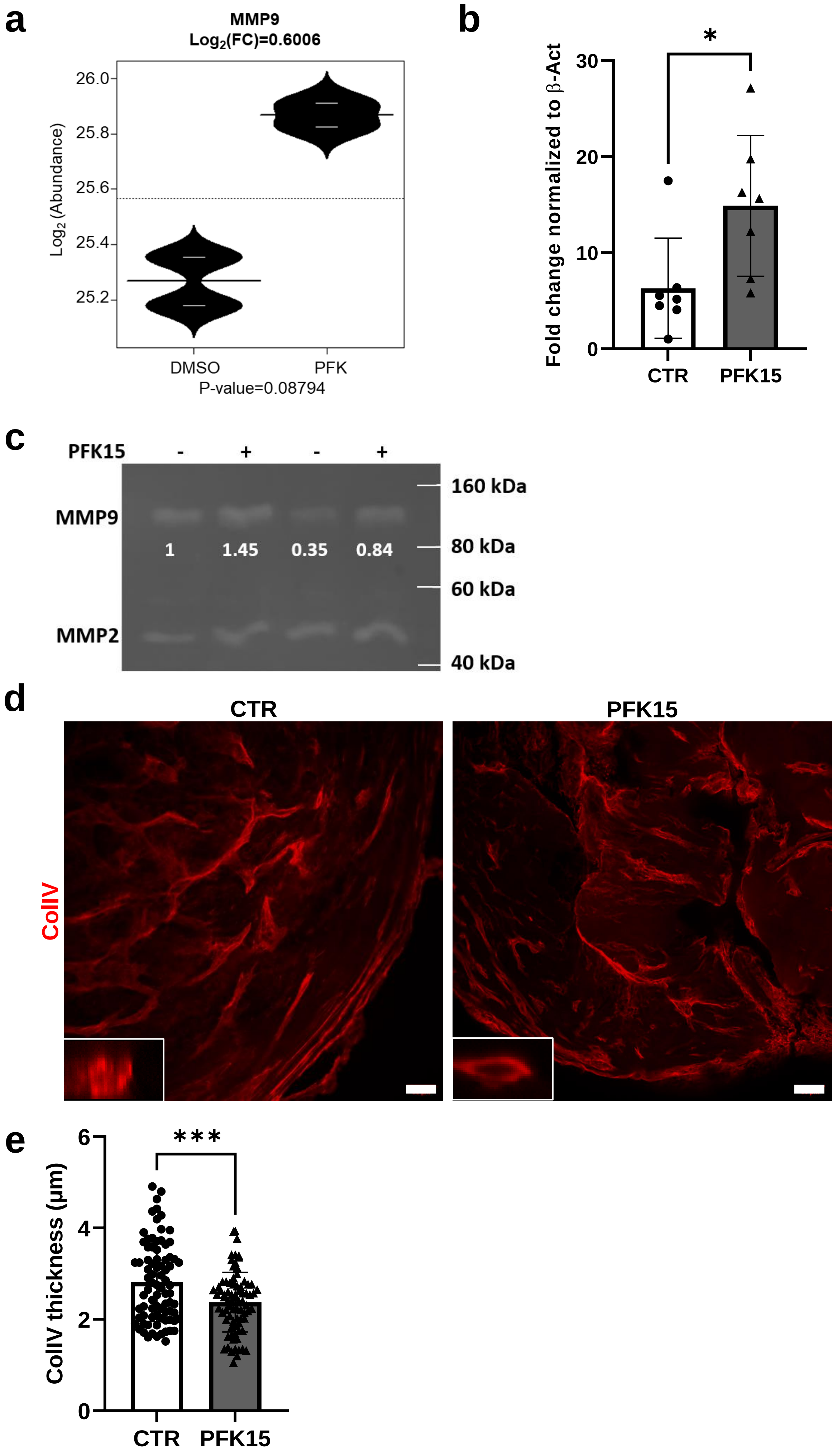

Supplementary Figure 7. Proteomic analysis of the BVO secretome (a) Differential expression of MMP9 in the BVO secretome as detected by proteomics, n=5 BVOs per pooled sample, 2 separate preparations. Statistical comparison was conducted using the Ebayes method of the limma package. Nominal p-value is displayed in beanplot while corrected for multiple testing p-value with the Benjamini-Hochberg method is provided in Supplemental Data 2. **(b)** MMP9 gene expression as assessed by qPCR, β-actin was used as a normalization control. n=7 BVOs per group, 3 separate preparations. Data are shown as mean ± SD. P values were calculated using a two-tailed Student's t-test. (*p=0.0270). **(c)** Enzymatic activity of gelatinases in the conditioned media of BVOs treated with DMSO (CTR) or PFK15 (2.5µM) for 24h, as assessed by zymography. n=5 BVOs per pooled sample, 2 separate preparations. Data were quantified using the ImageJ and normalised to total protein content, as measured by total spectra. **(d)** Deposition of CollV in BVOs following PFK15 treatment. Representative images of basement membrane, as detected by immunofluorescence confocal imaging for CollV in sections from BVOs treated with DMSO (CTR) or PFK15 (2.5 µM) 24h. Bar scales 50 µm. (**e**) Vessel cross-sections were used to quantify basement membrane thickening. N=80 cross-sections in single BVOs from 2 separate preparations per group. Values are presented as mean ± SD; P values were calculated using a two-tailed Student's t-test. (***p=0.0004).

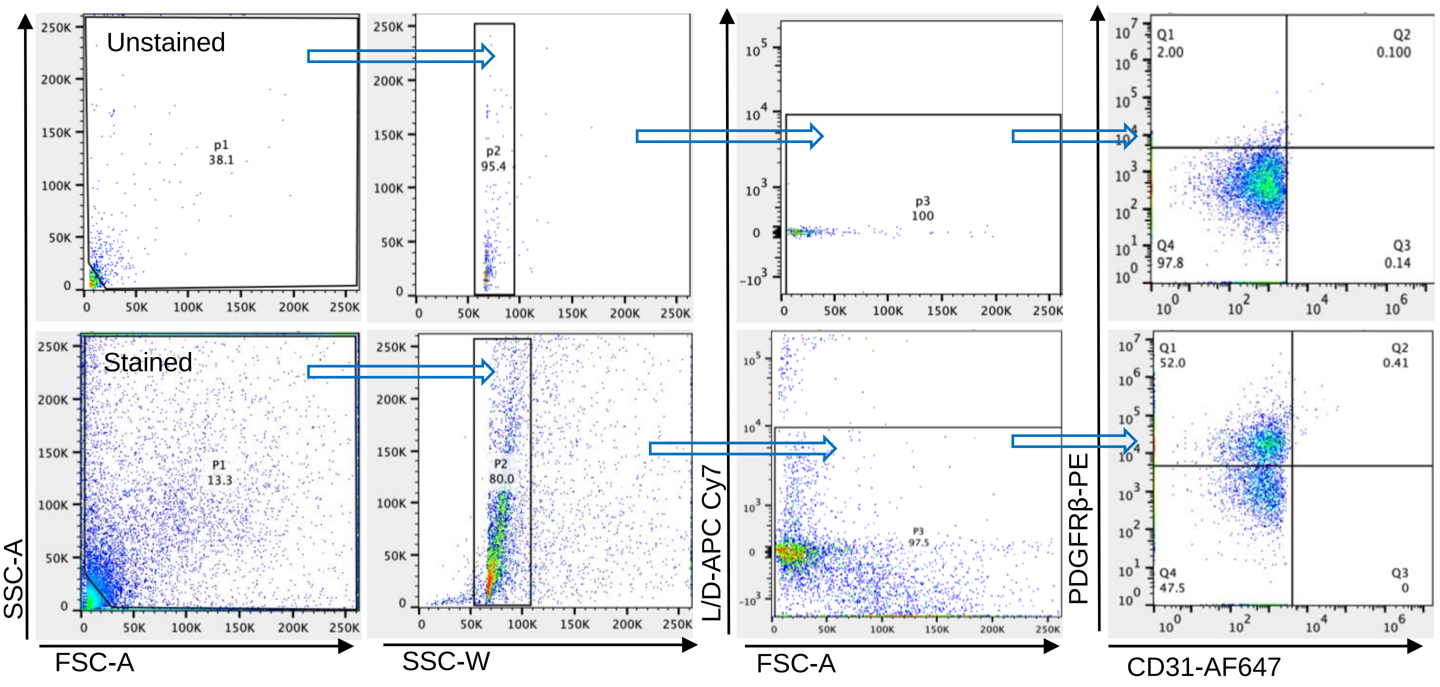

Supplementary Figure 8. FACS gating strategy. Gating strategy to determine cell percentage composition of BVOs in Figure 1e.

Supplementary Table S1

Antibodies used for Immunofluorescence staining

Primary antibody	Company Reference	Dilution IMF
**CD31**	R&D System AF806	1:100
**PDGFRβ**	Cell Signaling #3169	1:100
**Collagen IV**	Millipore AB769	1:200
**Oct4**	Thermo TA500035	1:100
**Nanog**	Sigma N3038	1:100
**CD144 (VE-cadherin)**	Millipore MABT134	1:200
**ZO1**	Santacruz Technologies sc-8147	1:100
**PDGFRα**	Abcam ab203491	1:1000
**YAP1**	NOVUS NB110-58358	1:500
**NG2**	Abcam ab86067	1:100
**Ki67**	Cell Signaling #9129S	1:100
**CC3 (Cleaved-Caspase 3)**	Cell Signaling #9661S	1:250

Secondary antibody	Company Reference	Dilution IMF
**Alexa-Fluor 488 Donkey anti-Sheep**	Invitrogen A11015	1:250
**Alexa-Fluor 488 Donkey anti-Mouse**	Invitrogen A21202	1:250
**Alexa-Fluor 488 Donkey anti-Rabbit**	Invitrogen A21206	1:250
**Alexa-Fluor 555 Donkey anti-Rabbit**	Invitrogen A31572	1:250
**Alexa-Fluor 633 Donkey anti-Mouse**	Invitrogen A21100	1:250
**Alexa-Fluor 647 Donkey anti-Rabbit**	Invitrogen A31573	1:250
**Alexa-Fluor 647 Donkey anti-Goat**	Jackson Immunolabs 705-606-147	1:250

Supplementary Table S2

Primary antibody	Company Reference	Dilution
**CD31-AlexaFluor647**	BD Biosciences, 558094	5µl/test
**CD140b-PE**	BD Biosciences, 558821	20µl/test
**CD144-FITC**	BD Biosciences, 560874	20µl/test
**CD45-FITC**	Invitrogen, 11-0459-41	5µl(0.25µg)/test
**CD90-PerCP/Cyanine5.5**	Biolegend, 328117	1µg/million cells
**CD73-BV650**	BD Biosciences, 742633	10µl/test
**CD44-PE**	BD Biosciences, 550989	20µl/test
**CD144-BV786**	BD Biosciences, 565672	5µl(0.25µg)/test
**Live/Dead-FVS780**	BD Biosciences, 565388	0.1µl/test

Supplementary Table S3

List of antibodies used for Western blot Analysis

Primary antibody	Company Reference	Dilution WB
**CD31**	Abcam ab28364	1:500
**CD144 (VE-cadherin)**	Millipore MABT134	1:2000
**KDR (VEGFR2)**	Cell Signaling #2479	1:1000
**eNOS**	BD Biosciences 610297	1:5000
**Oct4**	Thermo TA500035	1:1000
**GAPDH**	Santacruz Technologies sc-25778	1:1000
**Histone H3 (H-H3)**	Cell Signaling #9715S	1:1000
**YAP1**	NOVUS NB110-58358	1:500

Secondary antibody	Company Reference	Dilution WB
**Peroxidase AffiniPure Goat Anti-Mouse IgG**	Jackson Immunolabs 115-035-174	1:4000
**Peroxidase IgG Fraction Monoclonal Mouse Anti-Rabbit IgG**	Jackson Immunolabs 211-032-171	1:4000

Source Data file

Uncropped image of panel Supplementary Figure 6a.

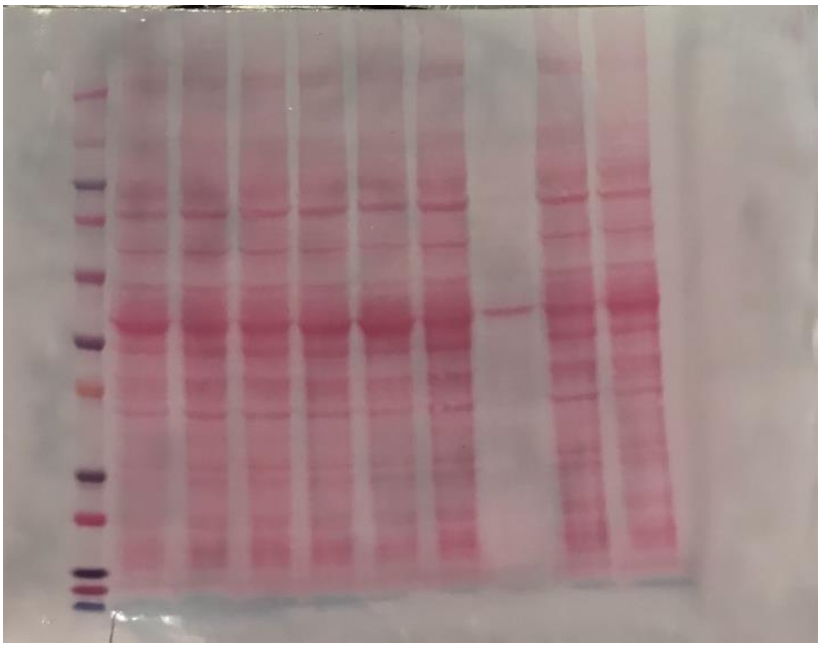

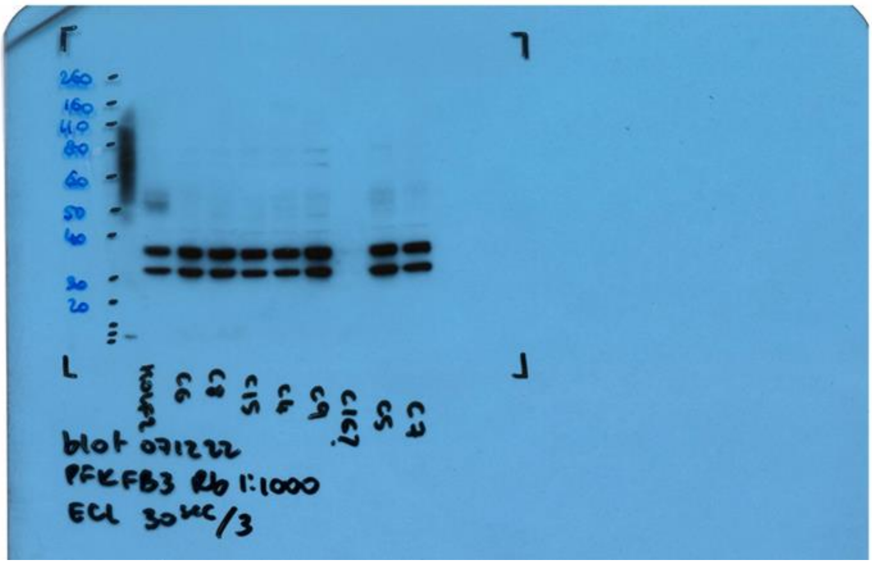

Source Data file

Uncropped image of panel Supplementary Figure 7c.

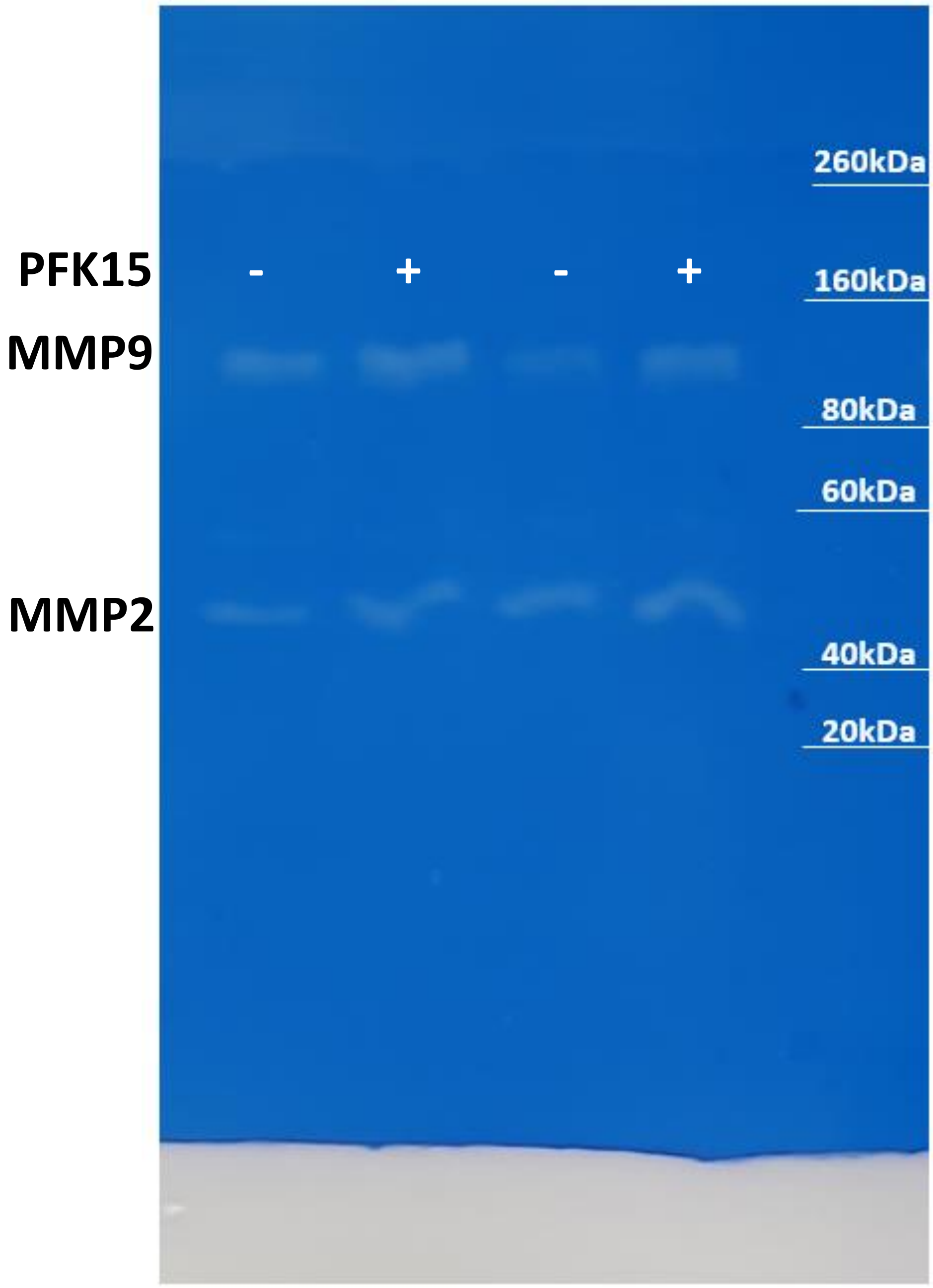

Supplement: Supplementary file 1 — Supplementary Information [file 41467_2023_41326_MOESM1_ESM.pdf]
