# Supplementary material for: Human blood vessel organoids reveal a critical role for CTGF in maintaining microvascular integrity
Source: Nat Commun. 2023 Sep 9;14:5552. doi: 10.1038/s41467-023-41326-2 (PMC10492781; doi:10.1038/s41467-023-41326-2)
Supplement: Supplementary file 2 — Description of Additional Supplementary Files [file 41467_2023_41326_MOESM2_ESM.pdf]

Description of Additional Supplementary Files

File Name: Supplementary Data 1
Description: Quantification of the metabolic profile of iPS-ECs following PFK15 treatment for 7h. Statistical comparisons were conducted using the Ebayes method of the limma package. Nominal p-values and corrected for multiple testing p-values with the Benjamini-Hochberg method are provided.

File Name: Supplementary Data 2
Description: Quantification of the proteomic dataset of the BVO secretome. Statistical comparisons were conducted using the Ebayes method of the limma package. Nominal p-values and corrected for multiple testing p-values with the Benjamini-Hochberg method are provided.

File Name: Supplementary Data 3
Description: Signal Peptide Predicted Scores using the signalP tool were used to classify the identified proteins in the proteomic dataset as secreted based in the presence of a signal peptide.

File Name: Supplementary Data 4
Description: The proteomic dataset of the BVO secretome, output of the identification software.

File Name: Supplementary Movie 1
Description: Confocal stack of a CTR BVO stained for CD31 (green) and PDGFR-β (red) and DAPI (blue). Bar scale 100 μm.

File Name: Supplementary Movie 2
Description: Confocal stack of a PFK15 treated BVO stained for CD31 (green), PDGFR-β (red) and DAPI (blue). Bar scale 100 μm.
