# Supplementary material for: Human blood vessel organoids reveal a critical role for CTGF in maintaining microvascular integrity
Source: Nat Commun. 2023 Sep 9;14:5552. doi: 10.1038/s41467-023-41326-2 (PMC10492781; doi:10.1038/s41467-023-41326-2)
Supplement: Supplementary file 9 — Reporting Summary [file 41467_2023_41326_MOESM9_ESM.pdf]

nature portfolio

Corresponding author(s): Anna Zampetaki

Last updated by author(s): Aug 1, 2023

Reporting Summary

Nature Portfolio wishes to improve the reproducibility of the work that we publish. This form provides structure for consistency and transparency in reporting. For further information on Nature Portfolio policies, see our Editorial Policies and the Editorial Policy Checklist.

Statistics

For all statistical analyses, confirm that the following items are present in the figure legend, table legend, main text, or Methods section.

n/a	Confirmed	
☐	☒	The exact sample size (*n*) for each experimental group/condition, given as a discrete number and unit of measurement
☐	☒	A statement on whether measurements were taken from distinct samples or whether the same sample was measured repeatedly
☐	☒	The statistical test(s) used AND whether they are one- or two-sided<br>*Only common tests should be described solely by name; describe more complex techniques in the Methods section.*
☒	☐	A description of all covariates tested
☐	☒	A description of any assumptions or corrections, such as tests of normality and adjustment for multiple comparisons
☐	☒	A full description of the statistical parameters including central tendency (e.g. means) or other basic estimates (e.g. regression coefficient) AND variation (e.g. standard deviation) or associated estimates of uncertainty (e.g. confidence intervals)
☐	☒	For null hypothesis testing, the test statistic (e.g. *F*, *t*, *r*) with confidence intervals, effect sizes, degrees of freedom and *P* value noted<br>*Give P values as exact values whenever suitable.*
☒	☐	For Bayesian analysis, information on the choice of priors and Markov chain Monte Carlo settings
☐	☒	For hierarchical and complex designs, identification of the appropriate level for tests and full reporting of outcomes
☒	☐	Estimates of effect sizes (e.g. Cohen's *d*, Pearson's *r*), indicating how they were calculated

Our web collection on statistics for biologists contains articles on many of the points above.

Software and code

Policy information about availability of computer code

Data collection	Immunostained samples were viewed and imaged using the Spinning Disk Confocal System (Nikon) and the Operetta CLS High-Content Analysis System (PerkinElmer). QPCR was performed using a ViiA 7 real-time PCR system. Flow cytometry data were acquired using a LSRFortessa Flow Cytometer analyzer (BD). Extracellular flux analyses studies were performed using a Seahorse XF24 analyzer (Agilent) and Cell nuclei were imaged using Li-cor Odyssey imager. Vascular network images were taken using a Nikon Eclipse TS100 microscope and Nikon DS-Fil camera. Peptide samples were injected and separated by an UltiMate3000 RSLCnano system (EASY-Spray C18 reversed-phase column, 75µm x 50cm, 2 µm, Thermo Fisher Scientific). The separated peptides were directly injected into an Orbitrap Q Exactive HF Mass Spectrometer (Thermo Fisher Scientific). For metabolomic analysis, a 1290 Infinity II ultrahigh performance liquid chromatography (UHPLC) system coupled to a 6546-quadrupole time-of-flight (Q-TOF) mass spectrometer (Agilent Technologies) were used. Luciferase activity was measured on an Infinite M200Pro (TECAN) plate reader.
Data analysis	ImageJ software (Fiji, version 1.53t) was used to quantify pericyte coverage and basement membrane thickness. Vessel Analysis Plugin on ImageJ was used to quantify vessel density and length. Viia7 software was used to analyse qPCR data. GraphPad Prism 9.0 software was used to prepare charts, t-tests and ANOVA tests. The Ebayes method of the limma package (version 3.56.2) was used for differential expression analysis of the proteomics data and corrections for multiple testing were conducted with the Benjamini-Hochberg method using python scripts. Beanplots and Volcano Plots were constructed using the Beanplots,Ggplot2 and Corrplot packages of the R programming environment, using R version 4.2.1. Network visualizations were conducted using Cytoscape tool (version 3.9.0) with protein-protein interaction networks being reconstructed from String web tool (version 11.5). Pathway and functional enrichment analysis were conducted using DAVID tool (version 2021). This analysis included pathway terms from Reactome Pathway Database (version 3.7), KEGG (release 101.0) and functional terms from Gene Ontology (release 2022-03). Transcriptional factor enrichment analysis was performed using the ChEA3 tool (version 3) using the ENCODE ChIP-sequencing data (update June 2017). Metabolite identification and feature annotation and were performed with MassHunter Profinder (version 10.0.2, Agilent Technologies) using our in-house curated metabolite library based on

metabolite standards (Sigma-Aldrich).
FACS data were analyzed using FlowJo software (Becton & Dickinson and Company).

For manuscripts utilizing custom algorithms or software that are central to the research but not yet described in published literature, software must be made available to editors and reviewers. We strongly encourage code deposition in a community repository (e.g. GitHub). See the Nature Portfolio guidelines for submitting code & software for further information.

Data

Policy information about availability of data

All manuscripts must include a data availability statement. This statement should provide the following information, where applicable:

- Accession codes, unique identifiers, or web links for publicly available datasets
- A description of any restrictions on data availability
- For clinical datasets or third party data, please ensure that the statement adheres to our policy

Source data are provided with this paper.

Proteomic raw data were analysed using Proteome Discoverer (version 2.4, Thermo Fisher Scientific) with MASCOT algorithm (version 2.6.0, Matrix Science) and UniProt/SwissProt human and bovine protein database version 2021_01, 26410 protein entries, https://www.uniprot.org/taxonomy/9606, https://www.uniprot.org/taxonomy/9913). Only proteins reported at the MatrisomeDB (https://matrisomedb.org/) plus some additional secreted proteins from our in-house generated database were considered. The signalP tool (version 5.0, https://services.healthtech.dtu.dk/services/SignalP-5.0/) was used to classify proteins as secreted. The limma package has been used to compare different phenotypes using the Ebayes algorithm and correcting for selected covariates. Beanplots and Volcano Plots were constructed using the Beanplots,Ggplot2 and Corrplot packages of the R programming environment, using R version 4.2.1. Network visualizations were conducted using Cytoscape tool (https://cytoscape.org/) with protein-protein interaction networks being reconstructed from String web tool (https://string-db.org/). Pathway and functional enrichment analysis were conducted using David tool (https://david.ncifcrf.gov/tools.jsp). This analysis included pathway terms from Reactome data repository (https://reactome.org/PathwayBrowser/) , KEGG (https://www.genome.jp/kegg/pathway.html) and functional terms from Gene Ontology (http://geneontology.org/docs/go-enrichment-analysis/). Transcriptional factor enrichment analysis was performed using the ChEA3 tool (https://maayanlab.cloud/chea3/) using the ENCODE ChIP-sequencing data (https://www.encodeproject.org/chip-seq/transcription_factor/).

All data generated or analysed during this study are included in the paper and in its supplementary information files. The mass-spectrometry proteomics data generated and analysed during the current study have been deposited to the ProteomeXchange Consortium via the PRIDE partner repository with the dataset identifier PXD041780 and https://doi.org/10.6019/PXD041780.

Research involving human participants, their data, or biological material

Policy information about studies with human participants or human data. See also policy information about sex, gender (identity/presentation), and sexual orientation and race, ethnicity and racism.

Reporting on sex and gender	This study did not involve human participants, their data or biological material.
Reporting on race, ethnicity, or other socially relevant groupings	This study did not involve human participants, their data or biological material.
Population characteristics	Not applicable to this study.
Recruitment	Not applicable to this study.
Ethics oversight	Not applicable to this study.

Note that full information on the approval of the study protocol must also be provided in the manuscript.

Field-specific reporting

Please select the one below that is the best fit for your research. If you are not sure, read the appropriate sections before making your selection.

☒ Life sciences ☐ Behavioural & social sciences ☐ Ecological, evolutionary & environmental sciences

For a reference copy of the document with all sections, see nature.com/documents/nr-reporting-summary-flat.pdf

Life sciences study design

All studies must disclose on these points even when the disclosure is negative.

Sample size	Sample size for every experiment is reported in the Figure legend or in the Methods section.<br>For quantitative analyses, sample size was determined based on similar studies in the field and no power/ sample size calculation was performed. For each experiment at least 3 biological replicates (BVO generation, iPS-EC differentiation) were performed based on previous publications (see references https://pubmed.ncbi.nlm.nih.gov/30651639/, https://pubmed.ncbi.nlm.nih.gov/32732889/, https://pubmed.ncbi.nlm.nih.gov/34923199/).

Data exclusions	No data was excluded from this study.
Replication	For all the assays, three independent experiments were performed . All the biological and technical samples were successful.
Randomization	Organoids from different preparations were randomly allocated into experimental groups.
Blinding	Blinding was not relevant to this study because all parameters applied were objective and no subjective assessment was involved.

Reporting for specific materials, systems and methods

We require information from authors about some types of materials, experimental systems and methods used in many studies. Here, indicate whether each material, system or method listed is relevant to your study. If you are not sure if a list item applies to your research, read the appropriate section before selecting a response.

Materials & experimental systems

n/a	Involved in the study	
☐	☒	Antibodies
☐	☒	Eukaryotic cell lines
☒	☐	Palaeontology and archaeology
☒	☐	Animals and other organisms
☒	☐	Clinical data
☒	☐	Dual use research of concern
☒	☐	Plants

Methods

n/a	Involved in the study	
☒	☐	ChIP-seq
☐	☒	Flow cytometry
☒	☐	MRI-based neuroimaging

Antibodies

Antibodies used

Primary Antibodies:
CD31, R&D System AF806
PDGFRβ, Cell Signaling #3169
Collagen IV, Millipore AB769
Oct4, Thermo TA500035
Nanog, Sigma N3038
CD144 (VE-cadherin), Millipore MABT134
ZO1, Santacruz Technologies sc-8147
PDGFRa, Abcam ab203491
YAP1, NOVUS NB110-58358
NG2, Abcam ab86067
Ki67, Cell Signaling #9129S
CC3 (Cleaved-Caspase 3), Cell Signaling #9661S
CD31-AlexaFluor647, BD Biosciences, 558094
CD140b-PE, BD Biosciences, 558821
CD144-FITC, BD Biosciences, 560874
CD45-FITC, Invitrogen, 11-0459-41
CD90-PerCP/Cyanine5.5, Biolegend, 328117
CD73-BV650, BD Biosciences, 742633
CD44-PE, BD Biosciences, 550989
CD144-BV786, BD Biosciences, 565672
CD31, Abcam, ab28364
KDR, Cell Signaling #2479
eNOS, BD Biosciences 610297
GAPDH, SantaCruz sc-25778
H-H3, Cell Signaling #9715S
Live/Dead-FVS780, BD Biosciences, 565388

Secondary Antibodies:
Alexa-Fluor 488 Donkey anti-Sheep, Invitrogen A11015
Alexa-Fluor 488 Donkey anti-Mouse, Invitrogen A21202
Alexa-Fluor 488 Donkey anti-Rabbit, Invitrogen A21206
Alexa-Fluor 555 Donkey anti-Rabbit, Invitrogen A31572
Alexa-Fluor 633 Donkey anti-Mouse, Invitrogen A21100
Alexa-Fluor 647 Donkey anti-Rabbit, Invitrogen A31573
Alexa-Fluor 647 Donkey anti-Goat, Jackson Immunolabs 705-606-147
Peroxidase-IgG Fraction Monoclonal Mouse Anti-Rabbit IgG, Jackson Immunolabs 211-032-171
Peroxidase-AffiniPure Goat Anti-Mouse IgG, Jackson Immunolabs 115-035-174

Validation

The specificity of the antibodies was provided by the manufacturers:

CD31, R&D System AF806. (https://www.rndsystems.com/products/human-cd31-pecam-1-antibody_af806)
PDGFRβ, Cell Signaling #3169. (https://www.cellsignal.com/product/productDetail.jsp?productId=3169)
Collagen IV, Millipore AB769. (https://www.merckmillipore.com/GB/en/product/Anti-Collagen-Type-IV-Antibody,MM_NF-AB769)
Oct4, Thermo TA500035. (https://www.thermofisher.com/antibody/product/OCT4-Antibody-clone-OTI9B7-Monoclonal/TA500035)
Nanog, Sigma N3038. (https://www.sigmaaldrich.com/GB/en/product/sigma/n3038)
CD144 (VE-cadherin), Millipore MABT134. (https://www.merckmillipore.com/GB/en/product/Anti-VE-cadherin-Antibody-clone-BV6,MM_NF-MABT134)
ZO1, Santacruz Technologies sc-8147. (https://www.scbt.com/p/zo-1-antibody-n-19)
PDGFRa, Abcam ab203491. (https://www.abcam.com/products/primary-antibodies/pdgfr-alpha-antibody-epr22059-270-ab203491.html)
YAP1, NOVUS NB110-58358. (https://www.novusbio.com/products/yap1-antibody_nb110-58358)
NG2, Abcam ab86067. (https://www.abcam.com/products/primary-antibodies/ng2-antibody-ab86067.html)
Ki67, Cell Signaling #9129S. (https://www.cellsignal.com/products/primary-antibodies/ki-67-d3b5-rabbit-mab/9129)
CC3 (Cleaved-Caspase 3), Cell Signaling #9661S. (https://www.cellsignal.com/product/productDetail.jsp?productId=9661)
CD31-AlexaFluor647, BD Biosciences, 558094. (https://www.bdbiosciences.com/en-gb/search-results?searchKey=558094)
CD140b-PE, BD Biosciences, 558821.(https://www.bdbiosciences.com/en-gb/search-results?searchKey=558821)
CD144-FITC, BD Biosciences, 560874.(https://www.bdbiosciences.com/en-gb/search-results?searchKey=560874)
CD45-FITC, Invitrogen, 11-0459-41. (https://www.thermofisher.com/antibody/product/CD45-Antibody-clone-HI30-Monoclonal/11-0459-42)
CD90-PerCP/Cyanine5.5, Biolegend, 328117. (https://www.biolegend.com/en-us/productstab/percp-cyanine5-5-anti-human-cd90-thy1-antibody-4515)
CD73-BV650, BD Biosciences, 742633. (https://www.bdbiosciences.com/en-us/search-results?searchKey=742633)
CD44-PE, BD Biosciences, 550989. (https://www.bdbiosciences.com/en-us/search-results?searchKey=550989)
CD144-BV786, BD Biosciences, 565672. (https://www.bdbiosciences.com/en-us/search-results?searchKey=565672)
CD31, Abcam, ab28364 (https://www.abcam.com/products/primary-antibodies/cd31-antibody-ab28364.html)
KDR, Cell Signaling #2479. (https://www.cellsignal.com/products/primary-antibodies/vegf-receptor-2-55b11-rabbit-mab/2479)
eNOS, BD Biosciences 610297. (https://www.bdbiosciences.com/en-gb/search-results?searchKey=610297)
GAPDH, SantaCruz sc-25778. (https://www.scbt.com/p/gapdh-antibody-fl-335)
H-H3, Cell Signaling #9715S.(https://www.cellsignal.com/products/primary-antibodies/histone-h3-antibody/9715)
Live/Dead-FVS780, BD Biosciences, 565388. (https://www.bdbiosciences.com/en-us/search-results?searchKey=565388)

Eukaryotic cell lines

Policy information about cell lines and Sex and Gender in Research

Cell line source(s)

KOLF2 iPS cells were obtained from the Wellcome Sanger Institute.
293T cells were purchased from LGC-ATCC (CRL-3216).
HUVECs were purchased from Promocell (C-12203).

Authentication

The IPS cell line was produced by the Wellcome Sanger Institute as part of the Human Induced Pluripotent Stem Cell Initiative (HIPSCI). Characterisation includes genotyping arrays, expression arrays, methylation arrays, RNA-seq, Exome-seq, proteomic mass-spectrometry, whole genome sequencing, and high content cellular phenotyping. IPS cells were further validated based on morphology, expression of pluripotent markers using PCR, Western blot and immunostaining. Exclusion of exogenous reprogramming factors was confirmed by Real Time PCR.

293T cells were authenticated by LGC-ATCC based on morphology and STR PROFILING (D3S1358: 15,17 TH01: 7,9.3 D21S11: 28,30.2 D18S51: 17,18 Penta_E: 7,15 D5S818: 8,9 D13S317: 12,14 D7S820: 11 D16S539: 9,13 CSF1PO: 11,12 Penta_D: 9,10 Amelogenin: X vWA: 16,19 D8S1179: 12,14 TPOX: 11 FGA: 23 D19S433: 18 D2S1338: 19).

HUVECs were authenticated by Promocell based on morphology and endothelial cell marker expression (CD31, vWF, Dil-Ac-LDL uptake positive).

Mycoplasma contamination

All cell lines were tested negative for mycoplasma contamination.

Commonly misidentified lines
(See ICLAC register)

No ICLAC line was used in this study.

Flow Cytometry

Plots

Confirm that:

☒ The axis labels state the marker and fluorochrome used (e.g. CD4-FITC).

☒ The axis scales are clearly visible. Include numbers along axes only for bottom left plot of group (a 'group' is an analysis of identical markers).

☒ All plots are contour plots with outliers or pseudocolor plots.

☒ A numerical value for number of cells or percentage (with statistics) is provided.

Methodology

Sample preparation	BVOs (n=7 per group) were mechanically dissociated using a scalpel and then incubated in Dissociation solution (1.7mg Dispase, 0.2mg Liberase and 0.1mg DNase per ml) in PBS for 20 min at 37°C. BVO solutions were passed up to 10 times through 21g needles. Approximately 50,000 single cells were resuspended in 100µl of FACS buffer (PBS containing 1% FBS) and stained with fluorescence-conjugated antibodies for 30 min at 4°C in the dark. The cells were washed in PBS and resuspended in 1% PFA in PBS. Data were acquired the following day using a Fortessa Flow Cytometer analyzer (BD) and analyzed using FlowJo software (Becton & Dickinson and Company).
Instrument	Flow cytometry data were acquired using a Fortessa Flow Cytometer analyzer (BD).
Software	Flow cytometry data were analyzed using FlowJo software (Becton & Dickinson and Company).
Cell population abundance	The abundance of PDGFRβ was 52%, CD31 positive cells 31%, CD73 positive cells 4.49%, CD4 positive cells 2.5%, CD45 positive cells 2.61%
Gating strategy	A gate was drawn around the population of interest excluding cells debris (P1)(SSC-A/FSC-A: 20k) and doublets (P2) (SSC-A/SSC-W: 60/90K). Dead cells were excluded in P3 using Live-Dead Gating/FSC-A. Positive cells were discriminated from negative ones based on the different markers gated from the unstained samples.

☒ Tick this box to confirm that a figure exemplifying the gating strategy is provided in the Supplementary Information.
